# An Eight-Gene Hypoxia Signature Predicts Survival in Pancreatic Cancer and Is Associated With an Immunosuppressed Tumor Microenvironment

**DOI:** 10.3389/fimmu.2021.680435

**Published:** 2021-05-20

**Authors:** Raefa Abou Khouzam, Shyama Prasad Rao, Goutham Hassan Venkatesh, Nagwa Ahmed Zeinelabdin, Stephanie Buart, Maxime Meylan, Manjunath Nimmakayalu, Stéphane Terry, Salem Chouaib

**Affiliations:** ^1^ Thumbay Research Institute for Precision Medicine, Gulf Medical University, Ajman, United Arab Emirates; ^2^ Bioinformatics Division, Yenepoya Research Center, Yenepoya University, Mangalore, India; ^3^ INSERM UMR 1186, Integrative Tumor Immunology and Cancer Immunotherapy, Gustave Roussy, EPHE, Faculty De médecine Univ. Paris-Sud, University Paris-Saclay, Villejuif, France; ^4^ Centre de Recherche des Cordeliers, Sorbonne Université, Inserm, Université de Paris, F-75006, Paris, France; ^5^ Graduate Program in Diagnostic Genetics and Genomics, School of Health Professions, MD Anderson Cancer Center, The University of Texas, Houston, TX, United States

**Keywords:** hypoxia, pancreatic cancer, prognostic, signature, tumor inflammation, microenvironment, immunosuppresive, immunotharapy

## Abstract

Intratumoral hypoxia is a widely established element of the pancreatic tumor microenvironment (TME) promoting immune escape, tumor invasion, and progression, while contributing to treatment resistance and poor survival. Despite this critical role, hypoxia is underrepresented in molecular signatures of pancreatic ductal adenocarcinoma (PDA) and concurrent investigations into the hypoxia-immune status are lacking. In this work a literature-based approach was applied to derive an eight-gene hypoxia signature that was validated in fourteen cancer cell lines and in a cohort of PDA. The eight-gene hypoxia signature was significantly associated with overall survival in two distinct PDA datasets and showed independent prognostic value in multivariate analysis. Comparative analysis of tumors according to their hypoxia score (high versus low) determined that tumors with high hypoxia were significantly less enriched in cytotoxic T-cells, and cytolytic activity. In addition, they had lower expression of cytokines and tumor inflammatory markers, pointing to the signature’s ability to discern an immune “cold”, hypoxic TME. Combining the signature with an immune metric highlighted a worse survival probability in patients with high hypoxia and low immune reactivity, indicating that this approach could further refine survival estimates. Hypoxia as determined by our signature, was significantly associated with certain immune checkpoint inhibitors (ICI) biomarkers, suggesting that the signature reflects an aspect of the TME that is worth pursuing in future clinical trials. This is the first work of its kind in PDA, and our findings on the hypoxia-immune tumor contexture are not only relevant for ICI but could also guide combinatorial hypoxia-mediated therapeutic strategies in this cancer type.

## Introduction

Pancreatic ductal adenocarcinoma (PDA) remains among the deadliest solid tumors, with a maximum estimated five-year survival rate of 10% in metastatic disease and up to 30% for resectable tumors (1). While much has been uncovered about the genetic background and progression dynamics of PDA, it continues to show resistance to current treatment regimens, including the recently introduced immune checkpoint inhibitors (ICI) (2–6). Hypoxia afflicts half of PDAs and has been associated with aggressive and metastatic tumors that are radio- and chemo-resistant (6–15). Moreover, hypoxia acts on various immune cells to promote an immunosuppressive tumor microenvironment (TME), pointing to its involvement as a potential underlying cause for the failure of ICI in PDA (4, 16, 17). The hypoxic phenotype, however, has been poorly represented in integrative studies that have put forth various PDA subgroups with distinct phenotypic, prognostic and predictive fractions based on variations in genetic background and cellular components (18–20). Identifying the degree of hypoxia in PDA and its interplay with the immune contexture of the TME could open the door to novel treatment combinations with enhanced patient selection strategies.

Hypoxia affects multiple hallmarks of PDA progression, including inhibition of apoptosis, induction of epithelial to mesenchymal transmission (EMT) and cancer stem cell-like properties, as well as reprogramming metabolic processes to sustain tumor survival (6). The drop in oxygen partial pressure indicative of hypoxia results in the stabilization of hypoxia inducible factors (HIFs), namely the ubiquitously expressed HIF-1α and the cell-type specific HIF-2α (21). HIF-1α, the master regulator of hypoxia, modulates pathways that are vital to PDA carcinogenesis and has been correlated with poor patient survival (22–26). While the pro-tumorigenic effect of hypoxia in PDA cannot be contended, some studies have highlighted a tumor suppressor role for HIFs, implicating them in having a protective function against tumor formation (27–30). It is important to note that such studies have focused on epithelial HIF expression, giving little credit to the impact of HIF signaling within the TME (31). The contradictory results could be context dependent, varying based on degree of hypoxia, tumor heterogeneity and the molecular background of the tumors (31). Clearly, more elaborative work is required to achieve a complete understanding of HIFs role in PDA carcinogenesis (31). Nonetheless, the application of strategies that target HIF-1α induced genes in combination with the chemotherapeutic agent gemcitabine enhanced its efficacy (11, 14). Targeting LOX, a driver of metastasis, in combination with gemcitabine significantly increased survival and reduced metastases in PDA-bearing KPC (*Pdx1-Cre Kras^G12D/+^* *Trp53^R172H/+^*) mice, compared to gemcitabine alone (11). In addition, co-treatment of an orthotopic PDX model of PDA with gemcitabine and an inhibitor of CA9 (a key enzyme in pH homeostasis), significantly reduced tumor growth and extended survival in comparison with gemcitabine monotherapy (14). Other evidence of the positive impact of alleviating hypoxia includes the potentiation of radiotherapy in preclinical pancreatic cancer mouse models cotreated with a hypoxia activated prodrug having selective cytotoxicity in hypoxic regions (12, 13, 15). Therefore, delineating the degree of hypoxia in PDA could prove highly informative in establishing prognosis and guiding treatment trajectories.

Common detection methods for hypoxia include the application of immunohistochemistry for determining hypoxia-induced proteins, imaging techniques for visualizing hypoxia sensitive agents, and more recently, there has been the novel approach of incorporating hypoxia gene signatures (32–37). Given the broad impact of hypoxia on gene expression profiles of both tumor cells and the surrounding stroma, multigene prognostic signatures have been published that act as a proxy to this condition in distinct tumor types (33–38). With respect to PDA, hypoxia signatures were found to be enriched in patients exhibiting worse prognosis (11). In an independent study a hypoxia signature was associated with worse overall survival (OS) and progression free survival (PFS) in patients with primary tumor; however, associations were lost in the presence of other clinical covariates (9). Recently, a thirty gene hypoxia signature was similarly found to be associated with worse OS and PFS in PDA (38). Despite these findings, no hypoxia signature to date has been tested in pancreatic cancer cell lines and validated in multiple patient datasets.

The availability of a hypoxia metric acting as a surrogate to tumor hypoxia would allow the investigation of other key players in the TME, namely immune cell fractions and immune activation status. Hypoxia promotes an immunosuppressive phenotype in the TME of PDA (4, 16, 17, 39); therefore, identifying the collective hypoxic and immune nature of a tumor could provide a gateway for reprogramming the TME from a non-immunogenic, immune “cold”, to an immunogenic, immune “hot”, through the reversal of its hypoxic state. This could be especially pertinent for stimulating response to ICI (39–41). As opposed to the success experienced in other solid tumors, ICI has shown limited efficacy in advanced PDA, wherein single agent treatment with anti-CTLA4 (cytotoxic T-lymphocyte-associated protein 4) (2) and anti-PDL1 (Programmed death-ligand 1) (3, 4) achieved zero percent response, and combined treatment, less than five percent response (4). The maximum response (18.2%) was in patients with mismatch repair deficiency/high microsatellite instability (MSI-H) treated with anti-PD1 (Programmed cell death protein 1) (5); however, such tumors constitute less than one percent of PDA (42). Indeed, PDAs have been shown to harbor low tumor mutational burden (TMB), especially in comparison with certain cancer types (43). In addition, they have moderate neoantigen load (44), low T-cell infiltration and PD-L1 (45, 46), all of which are ordinarily used as markers of response to ICI (47). Clearly, other TME markers are required to enhance the response outcome of ICI in this cancer type. Recently, a four-chemokine signature that could inform on a functional effector T-cell antitumor reactivity in the microenvironment of primary and metastatic PDA was suggested as an alternate indicator of response for immunotherapeutic strategies (48). Nonetheless, no prognostic associations were made, and hypoxia, a key element of the TME, was not investigated in the context of this signature.

In this study, we derived an eight-gene *in vitro* hypoxia gene signature and validated its application as a prognostic marker in two independent PDA datasets. We showed that the hypoxic state of PDA as determined by the signature is also indicative of an immunosuppressed TME, with a seemingly global mutual exclusivity between hypoxia and immune reactivity. In that setting, the combination of a dual hypoxia and immune metric identified a subset of patients with high hypoxia and low immune reactivity as having worse survival outcomes than those with low hypoxia, suggesting that this approach could further refine survival estimates and treatment recommendations in PDA, especially in the context of ICI.

## Materials and Methods

### Patient Datasets

Two independent datasets of PDA were included in this study and referred to as PAAD Data-1 and PAAD Data-2 (**Table 1**). PAAD Data-1 consists of 164 patients from the TCGA (The Cancer Genome Atlas) Pan-Cancer study (49) with stage I or II PDA, without any reported distant metastasis, and with available expression data. cbioportal was accessed (https://www.cbioportal.org/) to download patient clinical data, MSI score and mutation count, as well as RNAseq data and reverse phase protein array (RPPA) protein abundance data for genes of interest. With respect to PAAD Data-2, it consists of 309 patients with stage I or II PDA, without any reported distant metastasis and with available expression data (50), which were downloaded from the E-MTAB-6134 accession on ArrayExpress (https://www.ebi.ac.uk/arrayexpress/). For PAAD Data-1, the cytolytic index was additionally obtained from a previously published work (45), which reported it as the geometric mean of expression of *GZMA* and *PRF1*, coding for granzyme A and perforin-1, produced by activated cytolytic CD8+ T cells (45). For PAAD Data-2, the cytolytic index was manually derived by directly calculating the geometric mean of the two genes.

**Table 1:** Clinical characteristics of hypoxia scored PDA patient cohorts.

Dataset Characteristics	Whole cohort (n)	Hypoxia score high (n)	Hypoxia score low (n)
PAAD Data-1 (TCGA cohort)	164	66	98
Gender			
Male	92	37	55
Female	72	29	43
Age			
< 64 years	67	33	34
≥ 64 years	97	33	64
AJCC Stage			
I	1	0	1
IA IB	4 15	2 4	2 11
IIA	26	9	17
IIB	116	51	65
NA	2	0	2
Grade			
G1	29	5	24
G2	86	40	46
G3	47	21	26
G4	1	0	1
NA	1	0	1
PAAD Data-2 (E-MTAB-6134)	309	136	173
Gender			
Male	179	79	100
Female	130	57	73
AJCC Stage			
IA	8	5	3
IB	13	5	8
IIA	58	29	28
IIB	231	97	134
Grade			
G1	117	51	66
G2	134	58	76
G3	48	23	25
NA	10	4	6
Molecular subtype			
Immune Classical	35	7	28
Pure Classical	90	37	53
Desmoplastic	80	27	53
Stroma Activated	70	39	31
Pure Basal-like	34	26	8

### Cell Lines and Culture Conditions

Fourteen cancer cell lines were included representing six distinct solid cancer types that were maintained using appropriate media (**Supplementary Table S1**) in a humidified 5% CO_2_ incubator (ESCO Cell Culture incubator, USA). Three independent experiments were conducted per cell line by seeding a specific number of cells to prevent overcrowding and confluence (**Supplementary Table S1**). Twenty-four hours post seeding, cells were either kept in the incubator for another 24 hours at 21% O_2_ (normoxic conditions) or moved to the Whitley H35 Hypoxystation (Don Whitley Scientific Limited, UK) at 1% O_2_ (hypoxic conditions). Hypoxic conditions were confirmed by determining expression of HIF-1α (**Supplementary Methods**).

### RNA Extraction and Reverse Transcription-Quantitative PCR (RT-qPCR)

For each cell line, RNA was extracted using the easy-BLUE Total RNA kit (iNtRON Biotechnology, Korea) as per the manufacturer’s instructions, from biological duplicates of three independent experiments. RNA was resuspended in 20µL DEPC-treated water (Invitrogen, ThermoFisher scientific, USA), and the concentration and purity measured with the Nanodrop 2000 (Thermo scientific, USA). Two micrograms RNA was reverse transcribed using the High-Capacity cDNA Reverse Transcription Kit with RNase Inhibitor (Applied Biosystems, ThermoFisher scientific, USA) following manufacturer’s recommendations. RT-qPCR reactions were conducted in triplicate using 20ng cDNA, validated TaqMan gene expression assays (**Supplementary Table S2**) and TaqMan Gene Expression MasterMix (Applied Biosystems, ThermoFisher scientific, USA) at the recommended concentrations. The qPCRs were run in 96-well plates (Applied Biosystems, ThermoFisher scientific, USA) using the 7500 Fast Real-Time PCR System (Applied Biosystems, ThermoFisher scientific, USA). Fold change between hypoxic and normoxic conditions was calculated by applying the 2^-ΔΔCt^ method (51), with *ACTB* and *18S* as reference genes. Quantification was not performed for genes with Ct values greater than 34, and expression status was determined by running qPCR products on an agarose gel (**Supplementary Methods**).

### Scoring Patients’ Tumors

Each dataset was scored separately to prevent bias related to the sequencing platform used. For the hypoxia score (HS), this was done by first considering the difference in expression of the eight genes from their respective median expression in the dataset. If gene expression was less than the median, it was given a gene score of -1, else it scored as 1. The HS of a patient then totals as the sum of the gene scores. Patients were categorized as HS high only if their HS was greater than zero, otherwise they were HS low. The same approach was followed for determining the immune score (IS), only with the eighteen genes of the tumor inflammation signature (52) (**Supplementary Table S3**). Tumors were grouped as IS high or IS low based on their variation from zero. With respect to the four-chemokine signature, the scoring was done following the approach used in the publication (48); where briefly, patients were grouped into four-chemokine -low, -intermediate or -high, based on variation from the median of all genes.

### Estimation of Immune Cell Fractions

The digital cytometer, CIBERSORTx, was employed to determine the immune cell fractions in the microenvironment of HS high and HS low tumors. This tool, accessed at the web portal (https://cibersortx.stanford.edu/index.php), deconvolutes bulk tissue gene expression data to designate the fractions present of 22 tumor infiltrating immune cells defined by the LM22 signature matrix (53). These immune cells are subtypes of natural killer cells, T cells, dendritic cells, macrophages, mast cells, and monocytes, as well as neutrophils and eosinophils. Expression data for PAAD Data-1 and PAAD Data-2, were uploaded separately on the web portal and the absolute proportions of each cell type in the mixture were obtained by applying the LM22 signature for 100 permutations, followed by bulk-mode batch correction.

### Statistical and Survival Analysis

Two tailed t-test or ordinary one-way ANOVA were applied based on the number of groups being compared and relevant graphs were generated using GraphPad Prism 9.0.0 (GraphPad Software, La Jolla, USA). Survival analysis, heatmaps with hierarchical clustering and Spearman correlation analysis were conducted using RStudio V1.3 (RStudio, PBC, USA). For survival analysis, the following packages were acquired: survival, survminer, and survivalROC. Kaplan-Meier survival plots with log-rank test were used to compare the various groups based on the probability of overall survival (OS), disease specific survival (DSS) and progression free survival (PFS) for PAAD Data-1, and probability of OS and disease-free survival (DFS) for PAAD Data-2. Prognostic factors in the datasets were identified by applying univariate COX proportional hazard (PH) analysis to survival endpoints; while the independent prognostic power of the signature was determined by applying multivariate COX PH analysis, including the hypoxia score and prognostic clinical/molecular covariates. P-values < 0.05 were considered statistically significant. The potential of the signature in predicting survival was determined by generating ROC (receiving operating characteristics) curves and examining the AUC (area under the curve). Heat maps were generated using the gplots package, and HCA used Euclidean distance and Ward’s method as the clustering algorithm.

## Results

### Deriving a Common Eight-Gene *In Vitro* Hypoxia Signature

**Figure 1:**
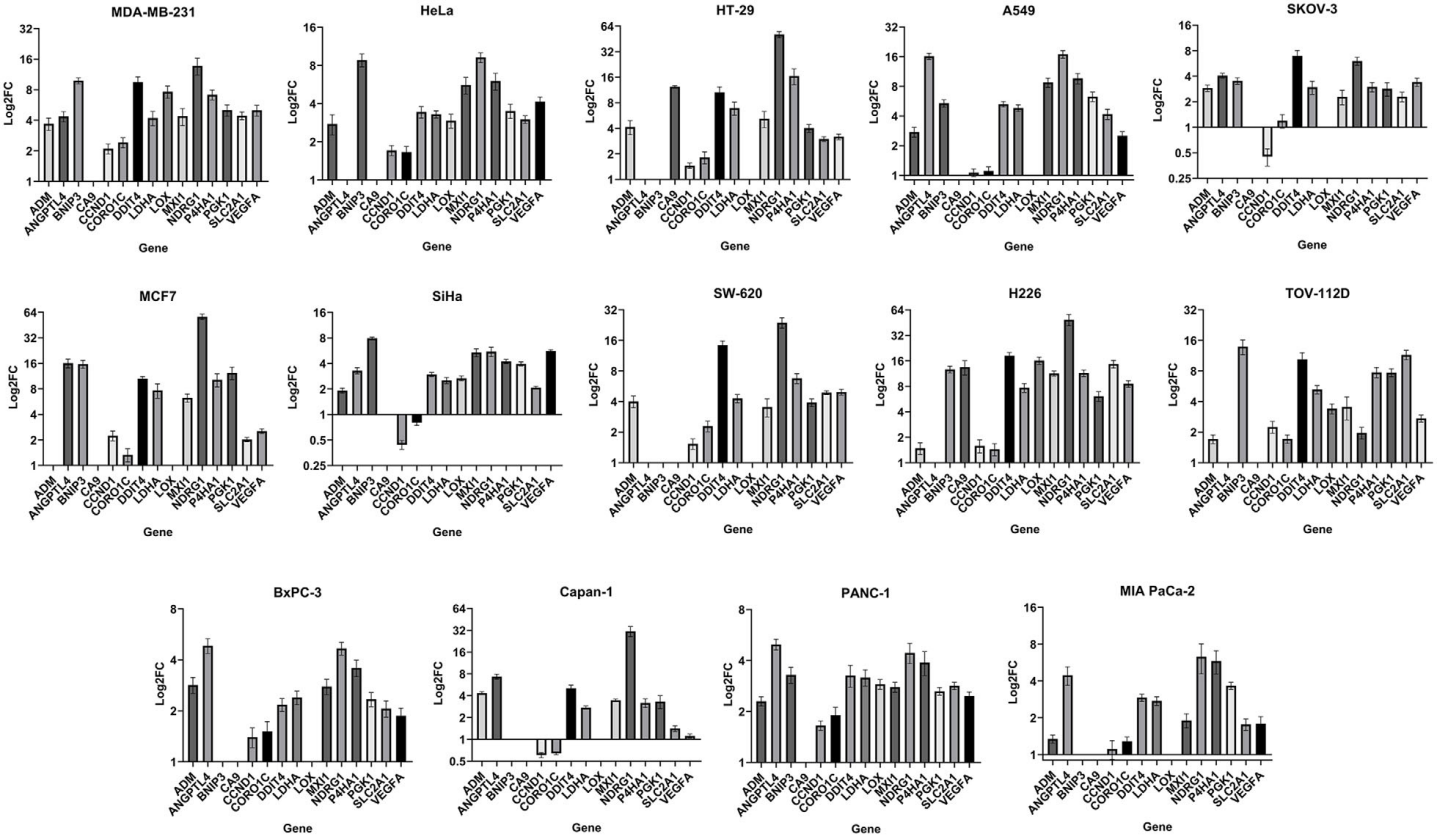
Gene expression fold change in fourteen cancer cell lines: LogFC (fold change) of gene expression in hypoxia (1% O_2_) relative to normoxia (21% O_2_) reported as mean ± SEM (standard error of mean) of three independent experiments. Absence of a FC value indicates absence of gene expression or negligible expression in normoxia, preventing normalization and calculation of FC (**Supplementary Figure S1B**).

As the foundation for deriving a common hypoxia signature, prognostic and predictive hypoxia gene signatures reported previously were first collected and compared to determine the unique and overlapping genes (**Supplementary Tables S4–6**). Only three of the reported signatures exhibited completely unique genes, while the rest shared a certain degree of overlap with other signatures, that ranged from around 7% to 100% (**Supplementary Table S4** **)**. In total 323 unique genes (**Supplementary Table S5**) and 75 common genes (**Supplementary Table S6**) could be mapped. To narrow down the list, fifteen testable genes were selected (**Table 2**) considering their recurrence rate in the signatures, the pan-cancer expression of the genes, as well as their implication in cancer-relevant pathways downstream of hypoxia (**Supplementary Methods**). The genes selected encompass pathways of cell metabolism (*LDHA, SLC2A1*), angiogenesis (*ANGPTL4, VEGFA*), EMT (*LOX*, *P4HA1*), cell growth and survival (*BNIP3, DDIT4*), among others (**Table 2**). To derive a common *in vitro* hypoxia gene signature, the sensitivity of the selected hypoxia-related genes to hypoxia was tested in fourteen selected cancer cell lines. These cell lines capture distinct tumor histopathological characteristics across six solid tumors of the lung, breast, colon and rectum, cervix, pancreas, and ovaries (**Supplementary Table S1**). Cells were exposed *in vitro* to either normoxia (21% oxygen) or hypoxia (1% oxygen) for 24 hours. These acute hypoxic conditions were confirmed by evaluating the expression of HIF-1α protein in all cell lines (**Supplementary Figure S1A**). Following RNA extraction, fold change of the fifteen genes was determined by RT-qPCR using specific TaqMan assays (**Figure 1**). Based on gene expression analysis, all fifteen genes showed some level of sensitivity to hypoxia in at least a fraction of the tested cell lines (**Figure 1**, **Supplementary Figure S1B**). Nonetheless, *BNIP3* and *LOX* were not expressed in all cell lines, while *ADM*, *ANGPTL4*, and *CA9* could not be quantified in all cell lines due to negligible expression (**Supplementary Figure S1B**). In addition, *CCND1* and *CORO1C* showed arbitrary variations in expression in response to hypoxia across the cell lines, ranging from no change to an increase or decrease in expression that was cell type dependent (**Figure 1**). Therefore, these seven genes were not included in the final signature. The final *in vitro* derived hypoxia gene signature consisted of eight genes that were quantifiable and showed at least a 2-fold increase in hypoxia, in more than half of the tested cell lines (**Supplementary Figure S1C**, **Table 2**). To determine the transferability of the signature, tumors of the TCGA pancreatic adenocarcinoma dataset, PAAD Data-1, were given a hypoxia score (HS) and the expression of PDA hypoxia-relevant genes involved in immune evasion, glycolysis, pH regulation and EMT were determined (6, 22–25). A significant increase in these genes could be confirmed in HS high tumors compared to HS low tumors, which was especially evident for the glycolysis genes, *ENO1* (p<0.001) and *HILPDA* (p<0.0001), as well as the immune evasion gene *ADAM10* (p<0.0001) (**Supplementary Figure S2A**). These genes were additionally significantly correlated with HS, and *ENO1* and *HILPDA* showed positive correlations with all the genes in the signature (**Supplementary Figure S2B**). Taken together, these results indicate that the score itself is acting as a metric for the hypoxic phenotype in patient tumors.

**Table 2:** The 15 selected genes.

**HGNC Symbol^#^**	**Gene Name**	**Function**	**Frequency**
***VEGFA***	vascular endothelial growth factor	Angiogenesis	10
***NDRG1***	N-myc downstream regulated 1	Stress response	10
***SLC2A1***	solute carrier family 2 member 1	Glucose metabolism	9
***P4HA1***	prolyl 4-hydroxylase subunit alpha 1	ECM remodeling	8
*ANGPTL4*	angiopoietin like 4	Angiogenesis	7
***LDHA***	lactate dehydrogenase A	Glycolytic metabolism	7
*BNIP3*	BCL2 interacting protein 3	Autophagy and apoptosis	7
***PGK1***	phosphoglycerate kinase 1	Glycolysis	6
***DDIT4***	DNA damage inducible transcript 4	Stress response	6
*ADM*	adrenomedullin	Angiogenesis	5
*CA9*	carbonic anhydrase 9	pH regulation	5
*LOX*	lysyl oxidase	ECM metabolism	4
***MXI1***	MAX interactor 1, dimerization protein	Transcriptional repressor	3
*CORO1C*	coronin 1C	Migration	2
*CCND1*	cyclin D1	Cell cycle	1

^#^In bold are genes included in the final eight-gene hypoxia signature.

ECM, extracellular matrix.

### Prognostic and Predictive Value of the Eight-Gene Hypoxia Signature in Two Independent PDA Datasets

**Figure 2:**
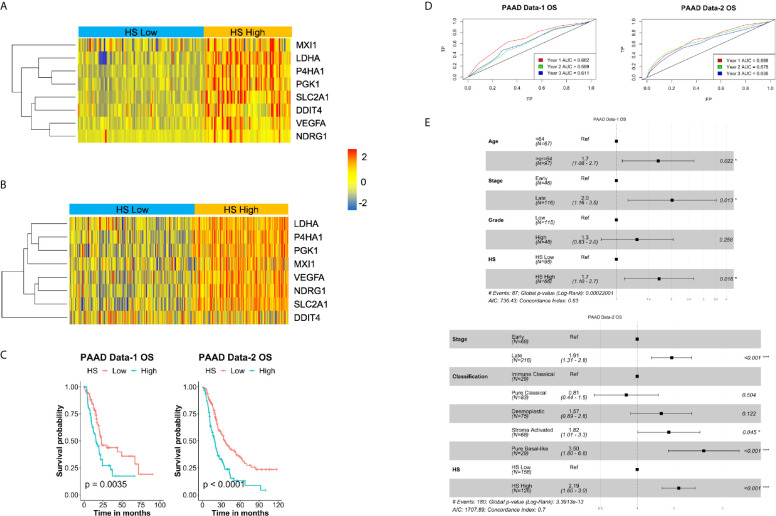
Prognostic and predictive value of hypoxia signature in pancreatic cancer: Heatmaps of the expression levels of the eight genes in the signature in hypoxia score (HS) high (in orange) and HS low (in blue) tumors in PAAD Data-1 **(A)** and PAAD Data-2 **(B)**. **(C)** Kaplan-Meier survival plots with log-rank test, comparing the groups divided based on hypoxia score in PAAD Data-1 and PAAD Data-2. **(D)** ROC curves depicting the predictive efficiency of the hypoxia signature on OS considering the 1-, 2-, and 3- years survival rates. **(E)** Multivariate COX PH forest plots showing hazard ratio of OS considering the 8-gene HS and other clinical covariates. P-value < 0.05 considered statistically significant; OS, overall survival; AUC, area under the curve; FP, false positive (1 - specificity); TP, true positive (sensitivity).

To determine the prognostic impact of the signature in PDA, two independent datasets were given hypoxia scores, PAAD Data-1 and PAAD Data-2 (**Table 1**, **Figures 2A, B**), subsequently survival parameters in the HS high and low tumors were compared. Significant association between the signature and survival was found in the two datasets; wherein patients with HS high tumors fared worse than those with HS low tumors with respect to their probability of OS (**Figure 2C**), as well as DSS, PFS and DFS (**Supplementary Figure S2C**). The strongest associations were for OS and DFS in PAAD Data-2 (p<0.0001). The predictive impact was assessed by carrying out ROC curve analysis, which showed that the signature achieves an AUC of 0.7 for predicting year-1 OS (**Figure 2D**) in both datasets, as well as DSS in PAAD Data-1 (**Supplementary Figure S2D**). Therefore, the eight-gene hypoxia signature represents a hypoxic phenotype that is associated with worse patient prognosis and the signature itself can predict early OS and DSS status with very-good distinguishing power in PDA.

### The Eight-Gene Hypoxia Signature Is an Independent Prognostic Marker in PDA

To confirm the risk associated with HS high tumors, both univariate and multivariate PH Cox analysis were applied considering the impact of HS alone, as well as HS in the presence of significant prognostic covariates (**Supplementary Table S7**). In keeping with the survival analysis results, there was a significant increase in hazard ratio (HR) in HS high tumors. The lowest being 1.7 (p=0.011) for PFS in PAAD Data-1 and the highest, 2.1 (p<0.001) for OS in PAAD Data-2 (**Supplementary Table S7**). For the multivariate PH Cox analysis, significant risk was associated with OS in HS high patients in both PDA datasets, even in the presence of other covariates (**Figure 2E**). HR in HS high tumors was 1.7 (p=0.016) in PAAD Data-1 and 2.19 (p<0.001) in PAAD Data-2. Similar results were achieved for DFS in PAAD Data-2, while borderline significance was noted for DSS in PAAD Data-1 (**Supplementary Figure S2E**). These findings indicate that the signature is not only associated with prognosis, but also acts as an independent prognostic factor, especially for OS, in pancreatic cancer. Moreover, when the eight-gene signature was compared to a recently published PDA hypoxia signature in PAAD Data-1 (38), the prognostic power of that signature was lost, while the eight-gene signature retained association with DSS and OS (**Supplementary Figure S3A**).

### Hypoxic Stress in PDA Is Associated With an Immune “Cold” TME

**Figure 3:**
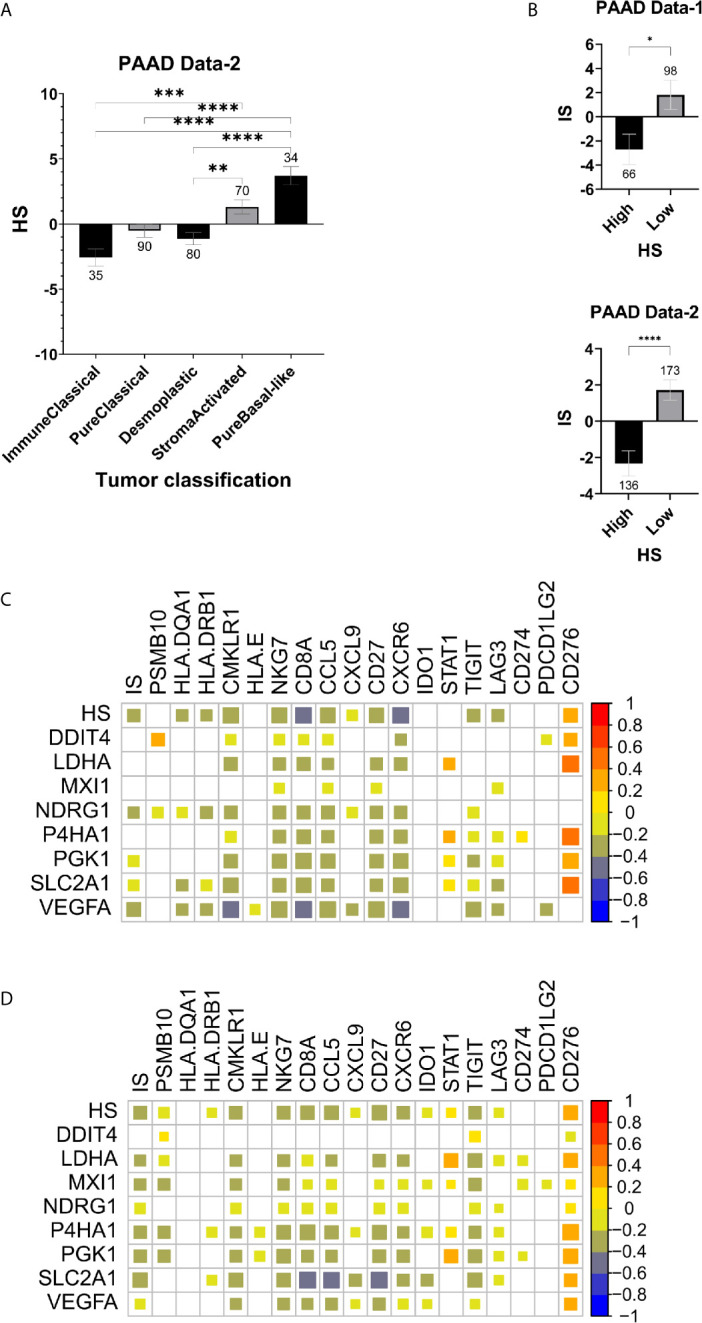
Hypoxia and immune markers in pancreatic cancer: **(A)** Mean SEM of hypoxia scores in tumors based on their molecular classification. **(B)** Mean SEM of immune scores (IS) based on the eighteen-gene tumor inflammation signature in HS high and low tumors. Spearman correlation plots between expression levels of the hypoxia signature and the tumor inflammation signature gene sets, as well as the immune score (IS) and hypoxia score (HS) in PAAD Data-1 **(C)** and PAAD Data-2 **(D)**. Strength of correlation reflected in the size of the colored square and white squares indicate no correlation **(C, D)**. Number of samples in each group is indicated above the error bars **(A, B)**. Significance determined by ordinary one-way ANOVA with Bonferroni correction for multiple testing **(A)** and two-tailed unpaired t-test analysis **(B)**. P-value < 0.05 considered statistically significant. *p-value > 0.01; **p-value < 0.0021; ***p-value < 0.0002; ****p-value<0.0001.

**Figure 4:**
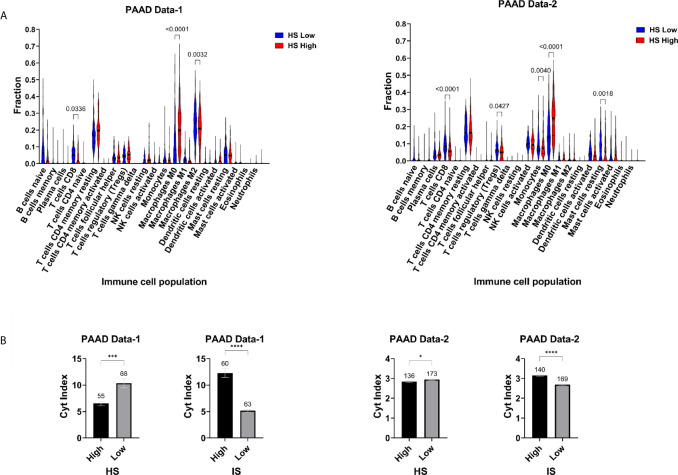
Hypoxia-immune landscape of pancreatic cancer: **(A)** Violin plots of immune cell fractions determined by CIBERSORTx in hypoxia score (HS) low and high tumors. **(B)** Mean SEM of Cytolytic (Cyt) index in HS high and low tumors and in IS high and low tumors. Number of samples in each group is indicated above the error bars. Significance determined by ordinary one-way ANOVA with Bonferroni correction for multiple testing **(A)** and two-tailed unpaired t-test analysis **(B)**. P-value < 0.05 considered statistically significant. *p-value > 0.01; **p-value < 0.0021; ***p-value < 0.0002; ****p-value < 0.0001.

The TME of PDA has been captured to some extent by molecular subtypes that integrate transcriptomic, genomic and molecular features. One such classification was used to stratify PAAD Data-2 into five subtypes of ImmuneClassical, PureClassical, Desmoplastic, StromaActivated and PureBasal-like (50). Comparing the hypoxia scores across these subtypes revealed significant differences in the distribution of hypoxia (**Figure 3A**). The biggest difference was between the ImmuneClassical and PureBasal-like subtypes (p<0.0001), with the highest scores concentrating in the basal-like subtype, while the lowest in the immune subtype. To investigate the seemingly mutual exclusivity between hypoxia, as presented by our signature, and the immune TME, as indicated by the molecular classification, an independent immune score (IS) was given to the same tumors in both datasets using a previously published tissue agnostic eighteen-gene tumor inflammation signature (45) (**Supplementary Table S3**). The degree of immune reactivity based on IS was investigated in HS high and low tumors and significant differences were found in PAAD Data-1 (p=0.013) and PAAD Data-2 (p<0.0001); wherein HS low tumors showed higher immune scores (**Figure 3B**). Spearman correlation analysis revealed significant inverse correlations between HS and IS (**Figures 3C, D**). In addition, the hypoxia score in both datasets showed negative correlations with ten genes in the tumor inflammation signature reflecting antigen presenting cell abundance (*HLA-DRB1* and *CMKLR1*), T-cell/NK-cell abundance (*NKG7*, *CD8A*), IFN activity (*CCL5*, *CXCL9*, *CD27*, *CXCR6*) and T-cell expressed exhaustion markers (*TIGIT*, *LAG3*). On the other hand, a positive correlation was obtained between HS and the inhibitory checkpoint *CD276* (B7-H3) (**Figures 3C, D**). These results support the impact of hypoxia on reshaping the tumor immune microenvironment. Immune cell fractions were subsequently determined using CIBERSORTx analysis, which reports on 22 immune cell types (**Figure 4A**). HS low tumors in PAAD Data-1 had significantly higher fractions of M2 macrophages, while in PAAD Data-2 they had significantly higher fractions of regulatory T cells, monocytes and resting mast, than the HS high tumors. Importantly, in both datasets HS low PDA showed significantly higher fractions of CD8+ T-cells than HS high tumors, which in turn exhibited significantly increased fractions of M0 macrophages. In addition, the cytolytic index, a measure of intratumoral cytolytic T-cell activity (20), was significantly higher in tumors with low hypoxia and in those with high IS in both PAAD Data-1 (p=0.0003; p<0.0001) and PAAD Data-2 (p=0.024; p<0.0001), compared to their respective high hypoxia, and low immune, counterparts (**Figure 4B**). This suggests a distinction in the immune cell milieu based on HS status of the tumor that could act to promote or quench its immunogenicity.

### A Combined Hypoxia-Immune Metric Enhances Survival Predictions in PDA

**Figure 5:**
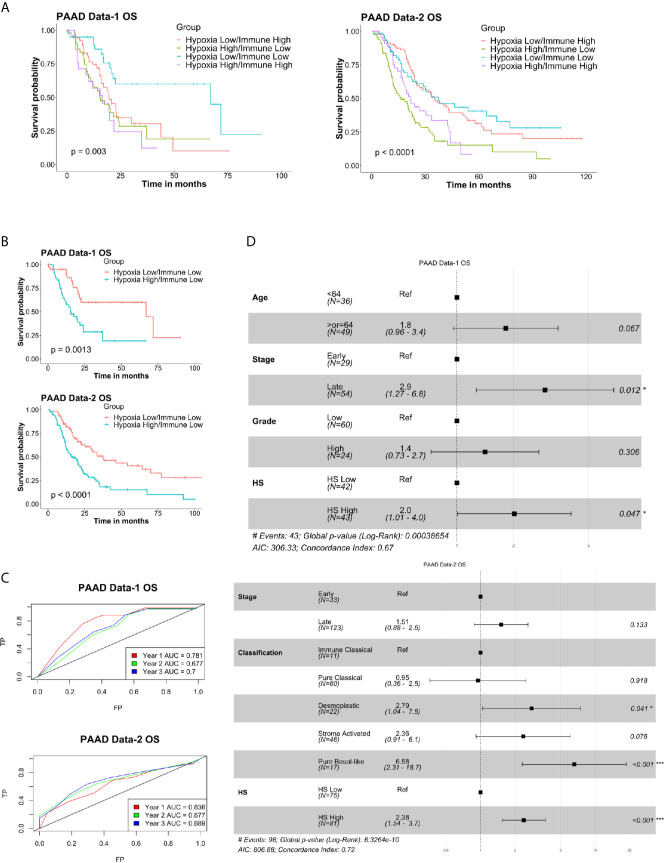
Prognostic and predictive efficiency of a combined hypoxia and immune classification in pancreatic cancer: **(A)** Kaplan-Meier survival plots with log-rank test, comparing the groups divided based on both hypoxia score and immune score. **(B)** Kaplan-Meier survival plots with log-rank test, comparing the two groups of Hypoxia Low/Immune Low and Hypoxia High/Immune Low. **(C)** ROC curves depicting the predictive efficiency of the hypoxia signature on OS considering the 1-, 2-, and 3- years survival rates in the two groups. **(D)** Multivariate COX PH forest plots showing hazard ratio of OS considering the 8-gene HS and other clinical covariates in the two groups. P-values < 0.05 considered statistically significant. OS, overall survival; AUC, area under the curve; FP, false positive (1 - specificity); TP, true positive (sensitivity).

To determine the prognostic efficiency of a combined hypoxia and immune classification, patients from each dataset were divided based on their immune and hypoxia scores into four distinct groups (**Supplementary Table S8**) and survival analysis was conducted. Significant difference in OS was detected in PAAD Data-1 (p=0.003) and PAAD Data-2 (p<0.0001) (**Figure 5A**), as well as differences in DSS (p=0.0028), PFS (p=0.0096) and DFS (p<0.0002) **(** **Supplementary Figure S4A**). Looking into how the groups varied, the worse probability for all survival endpoints was almost equally experienced by the groups having Hypoxia High, regardless of whether they were Immune Low or Immune High (**Figure 5A**, **Supplementary Figure S4A**). This could be due to the IS alone not being associated with survival (**Supplementary Figure S3B**). Nonetheless, the most protective effect was imparted by the Hypoxia Low/Immune Low microenvironment and a difference in survival was observed between Hypoxia High/Immune Low and Hypoxia Low/Immune Low subgroups in both datasets (**Figures 5A, B** **;** **Supplementary Figure S4A, B**). Further investigation into the impact of the hypoxia score on survival in these two groups showed a significant difference in OS in PAAD Data-1 (p=0.0013), and PAAD Data-2 (p<0.0001) (**Figure 5B**); as well as differences in DSS (p=00062), PFS (p=0015) in PAAD Data-1 and DFS (p=0.00075) in PAAD Data-2 (**Supplementary Figure S4B**). In addition, based on ROC curve analysis there was an increase in the AUC of prediction of survival by the HS in these two groups, reaching 0.7-0.8 for years 1, 2 and 3 OS, DSS and PFS in PAAD Data-1, as well 0.7 for year 2 and 3 OS and DFS in PAAD Data-2 (**Figure 5C**, **Supplementary Figure S4C**). Importantly, associations with DSS and DFS were retained in the presence of other clinical parameters (**Supplementary Figure S4D**), and higher hazard ratios imparted by hypoxia were obtained for OS in PAAD Data-1 (HR=2, p=0.047) and PAAD Data-2 (HR=2.38, p<0.001) (**Figure 5D**). These results indicate that in pancreatic cancer the absence of immune cells coupled with a hypoxic tumor microenvironment has an additional impact on survival probability.

### Association of PDA Hypoxic State With ICI Biomarkers

**Figure 6:**
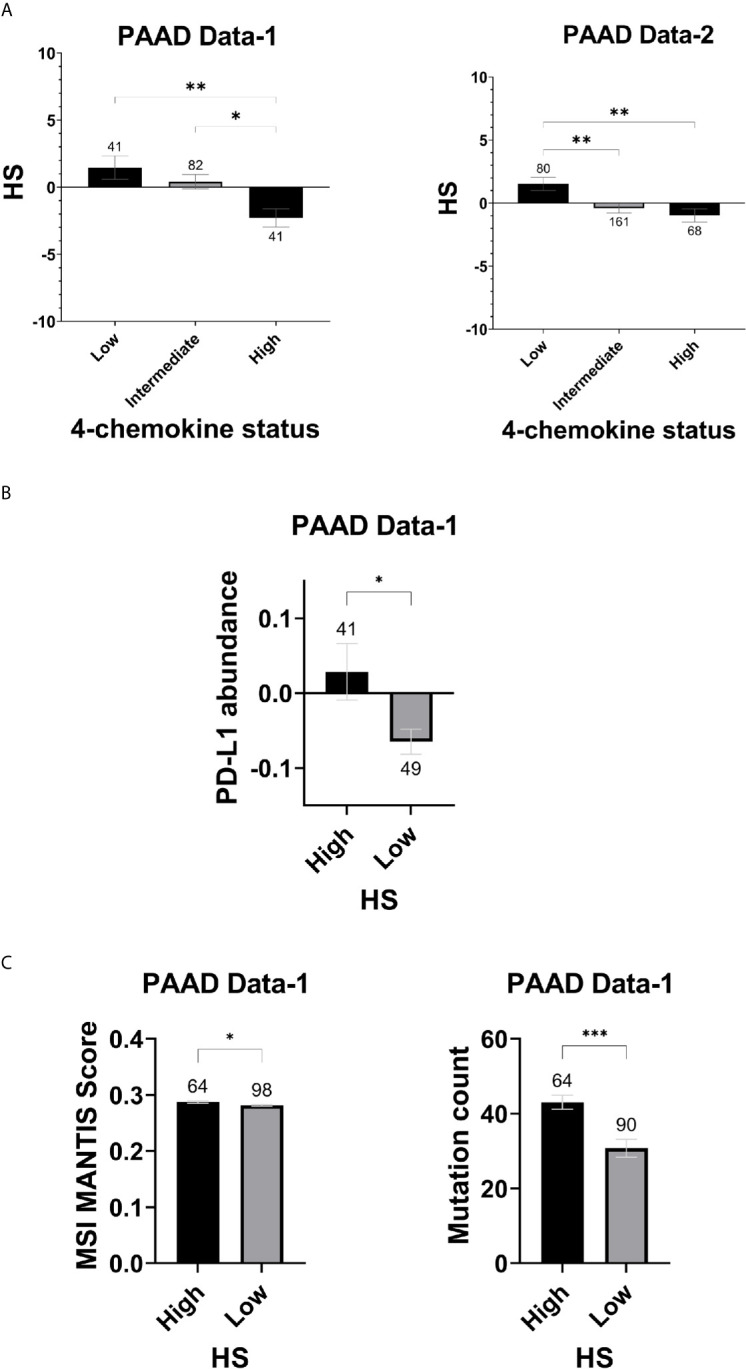
Hypoxia score and indicators of ICI in pancreatic cancer: **(A)** Mean SEM of hypoxia scores in tumors classified according to the four-chemokine signature. **(B)** Mean SEM of PDL-1 protein abundance levels based on RPPA in HS high and low tumors. **(C)** Mean SEM of MSI and Mutation count in HS high and low tumors. MSI scores are based on the MSI-calling tool, MANTIS. Significance determined by ordinary one-way ANOVA with Bonferroni correction for multiple testing **(A)** and two-tailed unpaired t-test analysis **(B, C)**. P-value < 0.05 considered statistically significant. Number of samples in each group is indicated above the error bars. *p-value > 0.01; **p-value < 0.0021; ***p-value < 0.0002; ****p-value < 0.0001.

To determine whether the eight-gene hypoxia signature is associated with other tumor features that could impact response to immune checkpoint inhibitors, differences in hypoxia scores were assessed in tumors grouped based on the four-chemokine signature (48). In addition, variations in PD-L1 status, mutation count and MSI were evaluated in HS high and low tumors of PAAD Data-1. Significantly higher hypoxia scores were present in tumors with low four-chemokine signature status in PAAD Data-1 (p=0.001) and PAAD Data-2 (p=0.001), indicating an inefficient cytotoxic immune response (**Figure 6A**). In parallel, HS high tumors had significantly higher protein abundance of PD-L1 (p=0.019), underlining an immunosuppressed TME (**Figure 6B**). Regarding, MSI, it was modestly enriched in HS high tumors compared to HS low tumors (p=0.023), while mutation counts were significantly higher in this group as well (p= 0.0002) (**Figure 6C**).

## Discussion

Characterizing the hypoxic state of pancreatic tumors and how it is interacting with the immune microenvironment could provide an alternate strategy in predicting patient survival and in guiding treatment trajectories, which could potentially alleviate the dismal prognosis of this disease. Increasing evidence has implicated hypoxia reversal with alleviation of resistance to chemo- and radio-therapy in PDA (11–15). As such, various clinical trials are being pursued to determine the impact of combination treatments with hypoxia-targeted approaches on patient response and survival (6). Among the completed clinical trials, a phase III trial found no survival advantage to the addition of a hypoxia targeting agent to gemcitabine in advanced pancreatic cancer (NCT01746979). Patient selection could be a key contributing factor to treatment success, and preemptively determining the degree of hypoxia in tumors could offer the possibility of enhancing treatment outcomes and prolonging survival. In this work we derived an eight-gene hypoxia signature with prognostic value for survival in PDA. We used the signature to investigate the immune component of this cancer as a function of its hypoxic state and to examine the impact of using a combined hypoxia and immune indicator in predicting patient survival.

The rise of prognostic and predictive hypoxia gene signatures in different cancer types, paved the way for our derivation of an eight-gene hypoxia signature. As opposed to other signatures, our signature was tested across multiple cancer types, including pancreatic cancer cell lines, that were exposed to hypoxic conditions *in vitro*. In this way, we managed to capture a common response to hypoxia, which we then applied in PDA patient samples. The relevance of the signature in PDA was confirmed by showing that tumors with high hypoxia exhibited significantly increased expression of hypoxia response genes pertinent in this cancer type. The presence of hypoxia as reflected by our signature was associated with worse OS in two independent datasets of PDA. This provides for a more robust signature than what has been previously published in this cancer (9, 38). Indeed, while a recent thirty-gene hypoxia signature was reported to be associated with worse OS and PFS in the TCGA dataset (38), this was not tested in an independent patient cohort. Moreover, when we compared survival parameters in tumors scored based on that signature, to those scored with our eight-gene signature, only ours maintained a significant association with OS and DSS. Given that the authors did not report the list of genes, we could not test their signature in PAAD Data-2. With respect to the other reported signature, it again was only tested in one dataset and the survival association found was lost in multivariate analysis, negating the need to test it in our cohorts (9). On the other hand, our signature was not only associated with OS, but was also independent of clinical covariates, namely grade and stage, in two independent cohorts of primary PDA. This points to a key conglomerate implication of the genes in our signature and the phenotype that they represent, in determining patient prognosis. Moreover, our evaluation of the signature *in vitro* allowed for the inclusion of only genes that can be quantified, which intensifies prospects of successfully scoring patient tumors using direct RT-qPCR based assays.

Molecular prognostication based on transcriptomes, genetic variations, and cellular components of pancreatic tumors have classified PDA into different subtypes (18–20). In this setting, hypoxia was found to dominate in the subtypes with worse prognosis (9, 20, 38). Recently, five transcriptomic subtypes that integrated elements from existing clusters, but additionally touching on the immune and inflammatory components, were put forth by a large multicentric study using gene expression profiles from FFPE samples of resectable pancreatic tumors (50). We found hypoxia to be significantly different among these subtypes of Immune classical, Pure classical, Desmoplastic, Stroma activated and Pure basal-like. In particular, the Pure basal-like, which was reported as imparting the worse prognosis in pancreatic cancer patients (50), had significantly higher hypoxia scores than the remaining subtypes. On the other hand, the lowest hypoxia scores were present in the immune classical subtype, which was reported as having the most protective effect on survival (50). This finding highlights the positive impact the absence of hypoxia can have on the tumor’s immune microenvironment in pancreatic cancer and how that can directly affect patient prognosis. Moreover, our signature maintained significant associations with both DFS and OS in the same pancreatic cancer dataset even in the presence of the molecular subtypes, suggesting that hypoxia, as captured by our signature, is representing additional factors that are independently affecting survival.

It is widely admitted that hypoxia in PDA deters the establishment of an effective anti-tumor response (4, 16, 17, 39–41). Using a validated, tissue agnostic eighteen-gene tumor inflammation signature (52), we showed that tumors with high hypoxia scores had significantly lower immune scores as indicated by that signature. Furthermore, the hypoxia score was inversely correlated with ten genes reflecting antigen presenting cell abundance, T-cell/NK-cell abundance, IFN activity and T-cell expressed exhaustion markers in both cohorts. On the other hand, a positive correlation was obtained between HS and *CD276*, which encodes the inhibitory checkpoint B7-H3. This finding underlines the strength of our signature in characterizing the hypoxic, immunosuppressive phenotype in PDA and makes for the first application of such a dualistic approach in pancreatic cancer. Moreover, the correlation between hypoxia and *CD276* (B7-H3) in PDA is novel and warrants in-depth investigation. While the immunologic function of B7-H3 remains controversial due to the lack of a known receptor (54), the checkpoint has shown low expression levels in normal cells, while being overexpressed in a variety of cancer types, including pancreatic cancer (54, 55). B7-H3 blockade using monoclonal antibodies and antibody-drug conjugates incited antitumor activity in B7-H3 positive tumors *in vivo*, and the combination of anti-PDL1 with B7-H3 has emerged as a promising strategy for the treatment of B7-H3-expressing non-small cell lung cancer (55–57). With respect to pancreatic cancer, high levels of B7-H3 are associated with worse survival outcome (55) and tumor progression (58), with *in vitro* work pointing to the involvement of B7-H3 in resisting gemcitabine-induced apoptosis in pancreatic cancer cell lines (59, 60). Moreover, targeting B7-H3 with the B7-H3.CAR (chimeric antigen receptor)-T cells derived from the monoclonal antibody effectively controlled tumor growth for up to 70 days post treatment in PDA xenograft models (57).

Further investigations into the tumor’s hypoxia-immune contexture revealed that tumors with low hypoxia had a significantly higher cytolytic index than their low hypoxia counterparts, which is indicative of the presence of a more active cytotoxic anti-tumor response. In line with this notion, less hypoxic tumors had significantly higher fractions of CD8+ T-cells, while more hypoxic tumors showed higher fractions of M0 macrophages in two independent datasets of pancreatic cancer. The presence of M0 macrophages in PDA has recently been shown to be associated with worse OS and relapse free survival, while the presence of CD8+ T-cells alluded to improved survival (61). In that respect, a large meta-analysis has also revealed that among tumor infiltrating lymphocytes, CD8+ T-cells show the strongest association with OS, and high infiltration improves patient prognosis (62). While we also found significantly higher fractions of certain immunosuppressive immune cell types in tumors with low hypoxia, namely M2 macrophages, resting mast cells, monocytes, and Tregs, these observations seem to be dataset-specific, and could point towards the involvement of other regulatory mechanisms that supersede hypoxia.

Given the complex interplay between the immune and hypoxic microenvironment, we delineated pancreatic cancer subgroups that could better predict survival considering both those elements of the TME. We stratified patients for the first time based on both their hypoxia and immune scores and observed significant differences in survival endpoints. While immune score alone was not associated with survival in the groups with high hypoxia, the hypoxia score was strongly associated with survival in the immune low groups. Indeed, hypoxia in these groups imparted increased risk, suggesting that in immune low patients especially, the presence of hypoxia has a detrimental impact on patient survival and could serve as an enhanced predictor of prognosis. It is important to note here that in the TCGA dataset, patients with hypoxia low and immune high tumors experienced worse survival than patients with hypoxia low and immune low tumors, which could be an indication of the distinct impact of the immune contexture on prognosis in tumors with low hypoxia. This difference was only seen in PAAD Data-1 and could also be due to inherent differences between the two datasets in terms of degree of tumor heterogeneity as well as sample collection and processing.

The importance of investigating the immune background based on the hypoxic status of pancreatic tumors is reflected in the failure to date of single agent immune checkpoint inhibitors from achieving the success experienced in other solid tumors (2–5). Indicators of ICI application and efficacy, including TMB, T-cell infiltration, neoantigen load, and PD-L1 expression (47), do not seem sufficient to achieve improved response. With this notion in mind, a four-chemokine signature has recently been proposed as an alternate indicator of response for immunotherapeutic strategies (48). In this work, we demonstrate that tumors with higher expression of our eight-gene hypoxia signature had significantly lower expression levels of the four-chemokine signature, accentuating the immunosuppressive role of hypoxia. Hypoxia contributes to immune evasion through various mechanism (63), including promoting the shedding of the major histocompatibility complex (MHC) class I polypeptide-related sequence A (MICA) (22, 64), as well as inducing the expression of checkpoint inhibitor, PD-L1, by cancer cells (65, 66), and myeloid derived suppressor cells (MDSCs) (66). Herein, we demonstrate that while there was no correlation between the hypoxia score and PD-L1 (*CD274*) at the transcript level based on the tumor inflammation signature, a significant increase in PD-L1 protein abundance could be seen in tumors with high hypoxia. The cellular source of PD-L1 cannot be pinpointed, nonetheless, a previous study that evaluated PD-L1 protein expression by immunohistochemical staining of pancreatic cancer tissue samples, revealed that tumors classified as PD-L1-positive had significantly less infiltrating T-cells and patients experienced worse overall survival (67). Furthermore, a recent meta-analysis of studies reporting PD-L1 expression by immunohistochemistry or PCR quantification, and PDA patient outcome, found that PDL-1 positivity/high expression was associated with significantly poorer survival (68). Therefore, our preliminary finding that hypoxia in a subset of pancreatic tumors seems to magnify PD-L1 expression, coupled with hypoxia being associated with worse survival outcomes in these patients, is in line with published findings. It is reasonable to premise that such patients with both high hypoxia and high PD-L1 could benefit from combinational treatment with hypoxia-targeted agents and PD1 or PDL-1 neutralizing antibodies. Such a strategy requires further investigation in PDA, especially since preclinical studies in other solid tumors on the effect of targeting or alleviating hypoxia in combination with ICI showed enhanced response rates and prolonged survival (39–41). In terms of other biomarkers of ICI, namely MSI and TMB, we could determine the association of hypoxia with the MSI MANTIS score and mutation count reported for PAAD Data-1. Such data was not available for PAAD Data-2. The MSI score was based on a computation method, MANTIS, which has been shown to have high sensitivity and specificity when tested in multiple cancer types with validated MSI status (69). The cutoff for this score is 0.4, making all the PDA tumors analyzed as microsatellite stable (70). Nonetheless, there was a significant increase of the MSI MANTIS score and mutation count in tumors classified as high hypoxia. This is in line with previous findings that hypoxia is associated with increased genetic instability and tumor mutational burden (71, 72). Therefore, it seems that the presence of hypoxia could be impacting various ICI relevant parameters and the signature could reflect an aspect of the TME that is worth pursuing in future ICI trials in PDA.

As with other studies with retrospective data, our findings require prospective validation. Regarding analysis conducted in a single dataset, they need additional replication in independent PDA cohorts. Given that the results were produced using data from patients with early-stage PDA, whether similar associations exist in late stage and metastatic disease warrants further investigation. In addition, the associations made between our signature and the immune microenvironment were based on transcriptomic data and could benefit from further *in vivo* validation using proteomic analysis and flow cytometry. Another caveat could be that the sample size in the comparison of survival parameters after grouping based on both the immune and hypoxia scores of the tumors was low and could have inflated the significance of the associations. We believe this to be unlikely, however, given the reproducibility of the finding for OS in two datasets.

Taken together, our findings point to the ability of the eight-gene signature to distinguish to some degree between immune active and deprived microenvironments that could be useful in predicting response to immunotherapy. In this context, our signature could potentially be used to determine the degree of tumor hypoxia, serving as a translational predictive marker to guide treatment trajectories. This is especially useful in recognizing the utility of novel hypoxia targeting and alleviation strategies and aiding their efficient and seamless translation to the clinic.

## Data Availability Statement

The original contributions presented in the study are included in the article/**Supplementary Material**. Further inquiries can be directed to the corresponding author.

## Ethics Statement

This work is in compliance with ethical standards. No human or animal subjects, or stem cells were used. No bio-safety/security issue was present. Standard laboratory practices and safety protocols were followed.

## Author Contributions

RA contributed to study design, data analysis and experimental work and wrote the manuscript. SP contributed to data analysis. GH gave suggestions on study design, discussed, and interpreted the data. SP and GH gave equal contributions to the work. NA contributed to experimental work and analysis. SB contributed to data discussion. MN contributed to conception. MM and ST contributed to data interpretation and critical review. SC designed and supervised the study. All authors contributed to the article and approved the submitted version.

## Funding

This work was funded by the Gulf Medical University.

## Conflict of Interest

The authors declare that the research was conducted in the absence of any commercial or financial relationships that could be construed as a potential conflict of interest.
