# Supplementary material for: An Eight-Gene Hypoxia Signature Predicts Survival in Pancreatic Cancer and Is Associated With an Immunosuppressed Tumor Microenvironment
Source: Front Immunol. 2021 May 20;12:680435. doi: 10.3389/fimmu.2021.680435 (PMC8173254; doi:10.3389/fimmu.2021.680435)
Supplement: Supplementary file 1 [file DataSheet_1.pdf]

Supplementary Methods

Gene selection

The gene selection process required the collection of hypoxia gene signatures with prognostic or predictive power that had been reported between 2006 and 2020 and that satisfied at least one of the following conditions: showed independent prognostic association / was validated in independent dataset(s) / was associated with validated markers of hypoxia (**Supplementary Table S4**). The genes were interrogated and compared among the signatures in search for overlapping and unique genes (**Supplementary Table S5-6**). To narrow down the list, fifteen testable genes were selected (**Table 2**). The first six genes were selected as they ranked highest in terms of their recurrence rate in signatures and encompassed pathways of cell metabolism (*LDHA*, *SLC2A1*), angiogenesis (*ANGPTL4*, *VEGFA*), EMT (*P4HA1*), and cell survival (*BNIP3*). From the second set of most frequent genes (those with a recurrence rate of six), two genes, *PGK1* and *DDIT4*, respectively contributing to cell metabolism and stress response were included. From the third set (those with a recurrence rate of five), two other genes, *ADM* and *CA9* were selected for their relevant roles in angiogenesis and pH regulation, respectively. The remaining four genes were included considering their involvement in cancer-modulating pathways of extracellular matrix remodeling (*LOX*), cell migration (*CORO1C*), cell cycle- (*CCND1*) and transcriptional- (*MXI1*) control (**Table 2**). All genes showed TCGA pan-cancer expression when interrogated on the human protein atlas (https://www.proteinatlas.org/).

Protein extraction and immunoblotting for HIF-1α

Following incubation in hypoxic or nomoxic conditions, protein was extracted using standard cold RIPA lysis buffer supplemented with 1X protease inhibitor cocktail (Sigma, USA) and quantified using the Pierce BCA protein estimation kit (ThermoFisher, USA) according to the manufacturer's protocol. In the same way, protein was extracted from MCF-7 treated with 250μM cobalt chloride (Sigma, USA) for 6 h in normoxia to induce HIF-1α. This protein extract was used as a positive control in the immunoblots. Thirty micrograms of total protein were separated on 10% SDS polyacrylamide gels and transferred to a Nitrocellulose membrane (Amersham, GE Healthcare Lifesciences, USA) for immunoblotting. Membranes were blocked with 5% bovine serum albumin (BSA) (Sigma, USA) and incubated first with the relevant primary antibody (Mouse β-Actin, (SantaCruz Biotechnology, USA) / Rabbit HIF-1α antibody (Cell Signaling Technologies, USA))

and then the appropriate HRP coupled secondary antibody (Goat anti-Mouse IgG, IgM (H+L) Secondary Antibody, HRP, (Invitrogen, ThermoFisher Scientific, USA) / Goat anti-Rabbit IgG (H+L) Secondary Antibody, HRP, (Invitrogen, ThermoFisher Scientific, USA)). In between and post incubations, membranes were washed as necessary with PBS 0.1% Tween 20 Detergent to minimize background. Visualization of the membranes was then conducted by first treating them with the SuperSignal West Pico PLUS Chemiluminescent Substrate kit (Thermo Scientific, USA) and capturing the signals with the iBright CL 1000 documentation system (Invitrogen, ThermoFisher Scientific, USA)

Agarose gel electrophoresis

Ten microliters of amplified qPCR products of *ADM* (76 bp), *CA9* (78 bp), *LOX* (77 bp), *BNIP3* (125 bp) and *ANGPTL4* (92 bp) were run on a 2% agarose gel for 30 min. Gels were prepared using low gelling agarose (Sigma, USA) dissolved in 1X TAE and 0.05% RedSafe Nucleic Acid Staining Solution (LiliF Diagnostic Products, iNtRON, Korea). Gel visualization was carried out using the Gel Doc XR+ Gel Documentation System (Bio-Rad Laboratories Inc, USA).
