# Supplementary material for: An Eight-Gene Hypoxia Signature Predicts Survival in Pancreatic Cancer and Is Associated With an Immunosuppressed Tumor Microenvironment
Source: Front Immunol. 2021 May 20;12:680435. doi: 10.3389/fimmu.2021.680435 (PMC8173254; doi:10.3389/fimmu.2021.680435)
Supplement: Supplementary file 2 [file DataSheet_2.pdf]

Table S1 Properties and culture conditions of cancer cell lines

Cancer	Cell line	Histopathological Feature	Source	Culture media[‡]	Cell number*
**Breast**	MDA-MB-231	Pleural effusion of metastatic triple negative adenocarcinoma	Addexbio Cat# C0006002 RRID: CVCL_0062	RPMI 1640	200'000
	MCF-7	Pleural effusion of metastatic adenocarcinoma	NCI-DTP Cat# MCF7, RRID: CVCL_0031	DMEM	400'000
**Cervical**	HeLa	Cervical adenocarcinoma, HPV+	ATCC Cat# CCL-2 RRID: CVCL_0030	DMEM	150’000
	SiHa	Cervical squamous cell carcinoma, HPV+	AddexBio Cat# C0008002 RRID: CVCL_0032	DMEM	200’000
**Colorectal**	HT-29	Colorectal adenocarcinoma	CLS Cat#330215 RRID: CVCL_0320	RPMI 1640	250’000
	SW-620	Lymph node metastasis of colorectal adenocarcinoma	CLS Cat#300466 RRID: CVCL_0547	DMEM	250’000
**Lung**	A549	Adenocarcinoma	NCI-DTP Cat# A549, RRID: CVCL_0023	RPMI 1640	250'000
	H226	Pleural effusion of metastatic squamous cell carcinoma	AddexBio Cat#C0016011 RRID: CVCL_1544	RPMI 1640	350’000
**Ovarian**	TOV-112D	Ovarian endometrioid adenocarcinoma	ATCC Cat# CRL-11731 RRID: CVCL_3612	DMEM	300’000
	SKOV-3	Ascites metastasis of ovarian serous cystadenocarcinoma	ATCC Cat# HTB-77, RRID: CVCL_0532	DMEM	250’000
**Pancreatic**	MIA PaCa-2	Pancreatic ductal carcinoma	ATCC Cat# CRL-1420, RRID: CVCL_0428	DMEM	300'000
	Capan-1	Liver metastasis of pancreatic ductal carcinoma	CLS Cat# 300143 RRID: CVCL_0237	RPMI 1640	450’000
	BxPC-3	Pancreatic ductal adenocarcinoma	ATCC Cat# CRL-1687 RRID: CVCL_0186	RPMI 1640	250’000
	PANC-1	Pancreatic ductal adenocarcinoma	ATCC Cat# CRL-1469 RRID: CVCL_0480	DMEM	400’000

[‡]All media has been supplemented with 10% FBS, 1% Sodium pyruvate and 1% Pen-Strep (Gibco, ThermoFisher Scientific, USA)

*Cell number seeded for experiments in normoxia and hypoxia

DMEM: Dulbecco's Modified Eagle Medium; RPMI: Roswell Park Memorial Institute

Table S2 TaqMan Gene Expression Assays

HGNC Symbol	Assay ID	Product size (bp)
VEGFA	Hs00900055_m1	59
NDRG1	Hs00608387_m1	54
SLC2A1	Hs00892681_m1	76
P4HA1	Hs00914594_m1	99
ANGPTL4	Hs01101127_m1	92
LDHA	Hs01378790_g1	66
BNIP3	Hs00969291_m1	125
PGK1	Hs99999906_m1	75
DDIT4	Hs01111686_g1	68
ADM	Hs00181605_m1	76
CA9	Hs00154208_m1	78
LOX	Hs00942483_m1	77
MXI1	Hs00365651_m1	102
CORO1C	Hs00902568_m1	62
CCND1	Hs00765553_m1	57
ACTB	Hs99999903_m1	171
18S	Hs99999901_s1	187

Table S3 Eighteen-gene tumor inflammation signature

Gene	Biological role
PSMB10	Antigen presenting cell abundance
HLA-DQA1	
HLA-DRB1	
CMKLR1	
HLA-E	T Cell/ NK Cell Abundance
NKG7	
CD8A	
CCL5	IFN Activity
CXCL9	
CD27	
CXCR6	
IDO1	
STAT1	
TIGIT	T Cell Exhaustion
LAG3	
CD274	
PDCD1LG2	
CD276	

Table S4 Prognostic and predictive hypoxia gene signatures

s.n.	Signature[#]	Prognostic / predictive capacity	Number of genes	Overlapping genes (%)
1	Chi JT et al., 2006 (1)	Breast cancer, ovarian cancer, lung cancer	123	17.89[ǂ]
2	Seigneuric R et al., 2007 (2)	Breast cancer; neuroblastoma	15	0.00
3	Winter SC et al., 2007 (3)	Breast cancer; head and neck squamous cell carcinoma; lung cancer	99	31.31
4	Hu Z et al., 2009 (4)	Breast cancer; lung cancer; glioma	13	53.85
5	Buffa FM et al., 2010 (5)	Breast cancer; lung cancer	51	56.86
6	van Malenstein H et al., 2010 (6)	Hepatocellular carcinoma	7	28.57
7	Fardin P et al., 2010 (7)	Neuroblastoma	32	78.13
8	Toustrup K et al., 2011 (8)	Head and neck squamous cell carcinoma*; soft tissue sarcoma	15	100.00
9	Halle C et al., 2012 (9)	Cervical cancer	31	54.84
10	Eustace A et al., 2013 (10)	Laryngeal cancer[§]	26	73.08
11	Ragnum HB et al., 2015 (11)	Prostate cancer	32	21.88
12	Fjeldbo S et al., 2016 (12)	Uterine cervical cancer	6	100.00
13	Buart S et al., 2017 (13)	Melanoma[§]	2	100.00
14	Loftus SK et al., 2017 (14)	Melanoma	10	30.00
15	Suh YE et al., 2017 (15)	Oropharyngeal squamous cell carcinoma	21	9.52
16	Yang L et al., 2017 (16)	Bladder cancer*	24	25.00
17	Yang L et al., 2018 (17)	Soft tissue sarcoma	24	79.17
18	Yang L et al., 2018 (18)	Prostate cancer; bladder cancer[§]	28	14.29
19	Ye IC et al., 2018 (19)	Basal subtype breast cancer	42	73.81
20	Dao Trong P et al., 2018 (20)	$IDH1^{mut}$ lower grade glioma	5	40.00
21	Lee JH et al., 2019 (21)	Colorectal cancer	4	0.00
22	Zou YF et al., 2019 (22)	Colorectal cancer	14	7.14
23	Deng F et al., 2020 (23)	Hepatocellular carcinoma	4	100.00
24	Lin W et al., 2020 (24)	Glioma	4	100.00
25	Mo Z et al., 2020 (25)	Lung adenocarcinoma	4	75.00
26	Sun J et al., 2020 (26)	Lung adenocarcinoma	16	12.50
27	Wang J et al., 2020 (27)	Breast cancer	14	35.71
28	Wang Z et al., 2020 (28)	Glioma	5	0.00
29	Karn T et al., 2020 (29)	Breast cancer[§]	3	66.67

[#] Prognostic and predictive hypoxia gene signatures since 2006 that satisfied at least one of the following conditions: showed independent prognostic association / was validated in independent dataset(s) / was associated with validated markers of hypoxia

* Both prognostic and predictive of therapeutic response

[§] Only predictive of therapeutic response

[ǂ] Based on the genes reported in the paper

$IDH1^{mut}$: Isocitrate Dehydrogenase 1 mutant

Table S5 Mapped unique genes in published prognostic and predictive hypoxia gene signatures

Unique Genes											
ACACA	*C4orf47*	*CSTB*	*ESRP1*	*GPI*	*ISG15*	*METTL22*	*PDIK1L*	*RHOC*	*SIK1*	*TFAP2C*	*TRMT5*
ACOT7	*CA12*	*CTSV*	*FABP5*	*GPN3*	*ITGA5*	*MIR210HG*	*PDLIM2*	*RIMKLA*	*SLC16A3*	*TFR2*	*TUBA1B*
ADAMTS4	*CAD*	*CXCL1*	*FAM117B*	*GRHPR*	*JAG2*	*MNAT1*	*PDSS1*	*RLF*	*SLC25A32*	*TFRC*	*TUBA1C*
ADORA2B	*CALD1*	*CXCL3*	*FAM120B*	*GRIN2D*	*KDM4A*	*MRPL13*	*PDZD11*	*RNF24*	*SLC2A14*	*TGFBI*	*TUBB2A*
AGTRAP	*CASP14*	*CXCL8*	*FAM13A*	*GRM3*	*KDM6A*	*MRPL14*	*PEX14*	*RNPS1*	*SLC2A1-AS1*	*THBD*	*TUBB6*
AHNAK2	*CASP6*	*CYB5R3*	*FAM83B*	*GSS*	*KIF20A*	*MRPL15*	*PFKL*	*RPL36A*	*SLC2A5*	*THBS1*	*TXLNG*
AK2	*CAV1*	*DAAM1*	*FBP1*	*GULP1*	*KIF4A*	*MS4A6A*	*PGAM4*	*RRAGD*	*SLC5A12*	*THBS4*	*TXNIP*
AKR7A2P1	*CCN1*	*DCBLD1*	*FBXO45*	*GYS1*	*KLF10*	*MTFP1*	*PGF*	*RRM2*	*SLC6A10P*	*TIMM23*	*UBE2D2*
AMH	*CCND1*	*DELEC1*	*FER1L4*	*H2AJ*	*KLF6*	*MTMR2*	*PGM1*	*RRP1B*	*SLCO1B3*	*TIMP2*	*UBR1*
ANG	*CCNG1*	*DNAH11*	*FGF21*	*HAUS2*	*LALBA*	*MTX1*	*PIN4*	*RUNX3*	*SLIRP*	*TIPARP*	*UCHL1*
ANKRD9	*CCNG2*	*DNAJC28*	*FLVCR2*	*HES2*	*LIF*	*MVD*	*PLAU*	*RUVBL2*	*SNTA1*	*TM4SF1*	*UNG*
APOL1	*CCT2*	*DONSON*	*FOSB*	*HJURP*	*LIFR*	*NAMPT*	*PLEKHG3*	*S100A10*	*SNX24*	*TMEM189*	*UTP11*
ASF1B	*CDC25C*	*DPM2*	*FOSL1*	*HLA-DRB5*	*LRP1*	*NCOA7*	*POU2AF1*	*S100A3*	*SOCS3*	*TMEM30B*	*VAPB*
ASPM	*CDCA4*	*DPYSL2*	*FOSL2*	*HMMR*	*LRP2BP*	*NDUFA4L2*	*PPAT*	*S100A7*	*SOD2*	*TMEM45A*	*VEZT*
ATF3	*CDCP1*	*DSC2*	*FOXM1*	*HMOX1*	*LRRC31*	*NEBL*	*PPM1J*	*SAV1*	*SORL1*	*TMPRSS11D*	*VHL*
ATP5MPL	*CDK18*	*DSP*	*FZD7*	*HOMER1*	*LRRC42*	*NIT1*	*PPP4R1*	*SCARB1*	*SPAG7*	*TMTC3*	*WDR45B*
B3GNT4	*CDK20*	*DTL*	*G6PD*	*HSPA1L*	*LYVE1*	*NME1*	*PRELID2*	*SCD*	*SPTB*	*TNIP1*	*WDSUB1*
B4GALT2	*CENPE*	*DTWD1*	*GAL*	*HSPB1*	*MAD2L2*	*NR0B2*	*PRSS53*	*SDAD1P1*	*SRPK1*	*TNS4*	*WNT6*
BACH1	*CENPU*	*E2F3*	*GAS6*	*ID3*	*MAFF*	*NR4A3*	*PSIP1*	*SDC1*	*SRPX*	*TPBG*	*XPNPEP1*
BCAR1	*CHCHD2*	*E2F6*	*GEM*	*IFI6*	*MAP7D1*	*NUDT15*	*PSMB7*	*SDC3*	*STBD1*	*TPD52*	*XPO5*
BCO1	*CLK3*	*EGR1*	*GEMIN2*	*IGF1R*	*MAPT*	*ORAI2*	*PSMD2*	*SEC61G*	*STC1*	*TPD52L2*	*XRCC6*
BIRC5	*CMTM3*	*EGR2*	*GJB6*	*IGFBP3*	*MAT1A*	*ORAI3*	*PSRC1*	*SEH1L*	*SYDE1*	*TPM4*	*YKT6*
BMS1	*CNIH4*	*EGR3*	*GLG1*	*IGFL2*	*MCL1*	*OTP*	*PTGFRN*	*SELE*	*SYNGR2*	*TRAF3*	*ZBTB44*
BNC1	*COL4A5*	*EIF2S1*	*GMFB*	*IL20RB*	*MCM2*	*PACS1*	*PUM1*	*SERPING1*	*TANC2*	*TRAM2*	*ZFP36*
BTG2	*COL4A6*	*ELAC1*	*GNAI1*	*IMMP2L*	*MCTS1*	*PAPPA*	*RAN*	*SH3GL3*	*TCAF2*	*TRAPPC1*	*ZNF292*
BUB3	*COL5A1*	*ENO2*	*GNRH1*	*INPP5J*	*MDM2*	*PAWR*	*RCL1*	*SHCBP1*	*TDG*	*TRIM9*	*ZWINT*
C16orf74	*CSRNP1*	*ERRFI1*	*GPC6*	*INSIG2*	*MEP1A*	*PCNA*	*RHOB*	*SIAH2*	*TEAD4*	*TRIP13*	

Table S6 Common genes and their frequency in published prognostic and predictive hypoxia gene signatures

Common genes	Count	Common genes	Count	Common genes	Count	Common genes	Count
NDRG1	10	*PFKFB4*	5	*PFKP*	3	*MIF*	2
VEGFA	10	*SLC2A3*	5	*PGAM1*	3	*PFKFB3*	2
SLC2A1	9	*BHLHE40*	4	*PLOD1*	3	*PKM*	2
P4HA1	8	*ERO1A*	4	*AK4*	2	*PNP*	2
ANGPTL4	7	*FAM162A*	4	*BTG1*	2	*PPARD*	2
BNIP3	7	*LOX*	4	*C4ORF3*	2	*PPFIA4*	2
LDHA	7	*MRGBP*	4	*CDKN3*	2	*PPP1R15A*	2
P4HA2	7	*PDK1*	4	*CORO1C*	2	*PSMA7*	2
ALDOA	6	*SLC16A1*	4	*CYP1B1*	2	*PVR*	2
DDIT4	6	*TPI1*	4	*DARS1*	2	*PYGL*	2
GAPDH	6	*AK3*	3	*DDIT3*	2	*RNASE4*	2
PGK1	6	*ALDOC*	3	*ECE2*	2	*S100A2*	2
ADM	5	*ANLN*	3	*EGLN1*	2	*SLC6A8*	2
ANKRD37	5	*ENO1*	3	*FGF11*	2	*SPAG4*	2
BNIP3L	5	*FUT11*	3	*ISG20*	2	*STC2*	2
CA9	5	*GBE1*	3	*JUNB*	2	*UPK1A*	2
EGLN3	5	*HK2*	3	*KDM3A*	2	*VLDLR*	2
HILPDA	5	*MRPS17*	3	*KRT17*	2	*ZNF395*	2
KCTD11	5	*MXI1*	3	*LDLR*	2		

Table S7 Univariate PH Cox analysis of potential prognostic parameters in PDA datasets

Cancer dataset	Clinical endpoint	Variable	Hazard ratio
**PAAD Data-1**	OS	HS (low *vs* high)	**1.9 (1.2-2.9) p = 0.004**
		Age (<64 *vs* ≥64)	**1.7 (1.1-2.6) p = 0.025**
		Gender (female *vs* male)	0.87 (0.57-1.3) p = 0.501
		Grade (G1+G2 *vs* G3+G4)	*1.5 (0.97-2.4) p = 0.068*
		Stage (I, IA, IB, IIA *vs* IIB)	**2.3 (1.3-4) p = 0.003**
	DSS	HS (low *vs* high)	**2 (1.2-3.2) p = 0.005**
		Age (<64 *vs* ≥64)	1.3 (0.81-2.1) p = 0.263
		Gender (female *vs* male)	0.81 (0.51-1.3) p = 0.394
		Grade (G1+G2 *vs* G3+G4)	*1.6 (0.95-2.6) p = 0.077*
		Stage (I, IA, IB, IIA *vs* IIB)	**3.1 (1.6-6) p = 0.001**
	PFS	HS (low *vs* high)	**1.7 (1.1-2.5) p = 0.011**
		Age (<64 *vs* ≥64)	1.1 (0.72-1.6) p = 0.708
		Gender (female *vs* male)	1 (0.96-1.5) p = 0.885
		Grade (G1+G2 *vs* G3+G4)	**1.7 (1.1-2.6) p = 0.014**
		Stage (I, IA, IB, IIA *vs* IIB)	**1.8 (1.1-3) p = 0.011**
**PAAD Data-2**	OS	HS (low *vs* high)	**2.1 (1.6-2.8) p < 0.001**
		Gender (female *vs* male)	1.2 (0.9- 1.6) p = 0.206
		Grade (G1+G2 *vs* G3)	1.3 (0.96-1.8) p = 0.084
		Stage (IA, IB, IIA *vs* IIB)	**1.7 (1.1-2.4) p = 0.007**
		Molecular subtype#	**1.5 (1.3-1.7) p < 0.001**
	DFS	HS (low *vs* high)	**1.8 (1.3-2.3) p < 0.001**
		Gender (female *vs* male)	1.3 (0.96- 1.7) p = 0.098
		Grade (G1+G2 *vs* G3)	**1.4 (1-1.8) p = 0.023**
		Stage (IA, IB, IIA *vs* IIB)	**1.7 (1.2-2.4) p = 0.001**
		Molecular subtype#	**1.4 (1.2-1.6) p < 0.001**

*In bold are significant p-values < 0.05. In italic are p-values of borderline significance. The 95% confidence interval is reported in brackets.

Immune classical used as reference *vs* pure classical, desmoplastic, stroma activated and pure basal-like

HS: hypoxia score; OS: overall survival; DSS: disease specific survival; PFS: progression free survival; DFS: disease-free survival

Table S8 Grouping PDA cohorts based on Immune score and Hypoxia score

Group	PAAD Data-1	PAAD Data-2
**G1: Hypoxia Low / Immune High**	56	91
**G2: Hypoxia High / Immune Low**	43	87
**G3: Hypoxia Low / Immune Low**	42	82
**G4: Hypoxia High / Immune High**	23	49
