# Supplementary material for: An Eight-Gene Hypoxia Signature Predicts Survival in Pancreatic Cancer and Is Associated With an Immunosuppressed Tumor Microenvironment
Source: Front Immunol. 2021 May 20;12:680435. doi: 10.3389/fimmu.2021.680435 (PMC8173254; doi:10.3389/fimmu.2021.680435)
Supplement: Supplementary file 3 [file DataSheet_3.pdf]

Supplementary References

1. Chi JT, Wang Z, Nuyten DS, Rodriguez EH, Schaner ME, Salim A, et al. Gene expression programs in response to hypoxia: cell type specificity and prognostic significance in human cancers. PLoS Med. 2006;3(3):e47.
2. Seigneuric R, Starmans MH, Fung G, Krishnapuram B, Nuyten DS, van Erk A, et al. Impact of supervised gene signatures of early hypoxia on patient survival. Radiother Oncol. 2007;83(3):374-82.
3. Winter SC, Buffa FM, Silva P, Miller C, Valentine HR, Turley H, et al. Relation of a hypoxia metagene derived from head and neck cancer to prognosis of multiple cancers. Cancer Res. 2007;67(7):3441-9.
4. Hu Z, Fan C, Livasy C, He X, Oh DS, Ewend MG, et al. A compact VEGF signature associated with distant metastases and poor outcomes. BMC Med. 2009;7:9.
5. Buffa FM, Harris AL, West CM, Miller CJ. Large meta-analysis of multiple cancers reveals a common, compact and highly prognostic hypoxia metagene. Br J Cancer. 2010;102(2):428-35.
6. van Malenstein H, Gevaert O, Libbrecht L, Daemen A, Allemeersch J, Nevens F, et al. A seven-gene set associated with chronic hypoxia of prognostic importance in hepatocellular carcinoma. Clin Cancer Res. 2010;16(16):4278-88.
7. Fardin P, Barla A, Mosci S, Rosasco L, Verri A, Versteeg R, et al. A biology-driven approach identifies the hypoxia gene signature as a predictor of the outcome of neuroblastoma patients. Mol Cancer. 2010;9:185.
8. Toustrup K, Sørensen BS, Nordsmark M, Busk M, Wiuf C, Alsner J, et al. Development of a hypoxia gene expression classifier with predictive impact for hypoxic modification of radiotherapy in head and neck cancer. Cancer Res. 2011;71(17):5923-31.
9. Halle C, Andersen E, Lando M, Aarnes EK, Hasvold G, Holden M, et al. Hypoxia-induced gene expression in chemoradioresistant cervical cancer revealed by dynamic contrast-enhanced MRI. Cancer Res. 2012;72(20):5285-95.
10. Eustace A, Mani N, Span PN, Irlam JJ, Taylor J, Betts GN, et al. A 26-gene hypoxia signature predicts benefit from hypoxia-modifying therapy in laryngeal cancer but not bladder cancer. Clin Cancer Res. 2013;19(17):4879-88.
11. Ragnum HB, Vlatkovic L, Lie AK, Axcrona K, Julin CH, Frikstad KM, et al. The tumour hypoxia marker pimonidazole reflects a transcriptional programme associated with aggressive prostate cancer. Br J Cancer. 2015;112(2):382-90.
12. Fjeldbo CS, Julin CH, Lando M, Forsberg MF, Aarnes EK, Alsner J, et al. Integrative Analysis of DCE-MRI and Gene Expression Profiles in Construction of a Gene Classifier for Assessment of Hypoxia-Related Risk of Chemoradiotherapy Failure in Cervical Cancer. Clin Cancer Res. 2016;22(16):4067-76.
13. Buart S, Terry S, Noman MZ, Lanoy E, Boutros C, Fogel P, et al. Transcriptional response to hypoxic stress in melanoma and prognostic potential of GBE1 and BNIP3. Oncotarget. 2017;8(65):108786-801.
14. Loftus SK, Baxter LL, Cronin JC, Fufa TD, Pavan WJ, Program NCS. Hypoxia-induced HIF1α targets in melanocytes reveal a molecular profile associated with poor melanoma prognosis. Pigment Cell Melanoma Res. 2017;30(3):339-52.
15. Suh YE, Lawler K, Henley-Smith R, Pike L, Leek R, Barrington S, et al. Association between hypoxic volume and underlying hypoxia-induced gene expression in oropharyngeal squamous cell carcinoma. Br J Cancer. 2017;116(8):1057-64.

16. Yang L, Taylor J, Eustace A, Irlam JJ, Denley H, Hoskin PJ, et al. A Gene Signature for Selecting Benefit from Hypoxia Modification of Radiotherapy for High-Risk Bladder Cancer Patients. Clin Cancer Res. 2017;23(16):4761-8.
17. Yang L, Forker L, Irlam JJ, Pillay N, Choudhury A, West CML. Validation of a hypoxia related gene signature in multiple soft tissue sarcoma cohorts. Oncotarget. 2018;9(3):3946-55.
18. Yang L, Roberts D, Takhar M, Erho N, Bibby BAS, Thiruthaneeswaran N, et al. Development and Validation of a 28-gene Hypoxia-related Prognostic Signature for Localized Prostate Cancer. EBioMedicine. 2018;31:182-9.
19. Ye IC, Fertig EJ, DiGiacomo JW, Considine M, Godet I, Gilkes DM. Molecular Portrait of Hypoxia in Breast Cancer: A Prognostic Signature and Novel HIF-Regulated Genes. Mol Cancer Res. 2018;16(12):1889-901.
20. Dao Trong P, Rösch S, Mairbäurl H, Pusch S, Unterberg A, Herold-Mende C, et al. Identification of a Prognostic Hypoxia-Associated Gene Set in IDH-Mutant Glioma. Int J Mol Sci. 2018;19(10).
21. Lee JH, Jung S, Park WS, Choe EK, Kim E, Shin R, et al. Prognostic nomogram of hypoxia-related genes predicting overall survival of colorectal cancer-Analysis of TCGA database. Sci Rep. 2019;9(1):1803.
22. Zou YF, Rong YM, Tan YX, Xiao J, Yu ZL, Chen YF, et al. A signature of hypoxia-related factors reveals functional dysregulation and robustly predicts clinical outcomes in stage I/II colorectal cancer patients. Cancer Cell Int. 2019;19:243.
23. Deng F, Chen D, Wei X, Lu S, Luo X, He J, et al. Development and validation of a prognostic classifier based on HIF-1 signaling for hepatocellular carcinoma. Aging (Albany NY). 2020;12(4):3431-50.
24. Lin W, Wu S, Chen X, Ye Y, Weng Y, Pan Y, et al. Characterization of Hypoxia Signature to Evaluate the Tumor Immune Microenvironment and Predict Prognosis in Glioma Groups. Front Oncol. 2020;10:796.
25. Mo Z, Yu L, Cao Z, Hu H, Luo S, Zhang S. Identification of a Hypoxia-Associated Signature for Lung Adenocarcinoma. Front Genet. 2020;11:647.
26. Sun J, Zhao T, Zhao D, Qi X, Bao X, Shi R, et al. Development and validation of a hypoxia-related gene signature to predict overall survival in early-stage lung adenocarcinoma patients. Ther Adv Med Oncol. 2020;12:1758835920937904.
27. Wang J, Wang Y, Xing P, Liu Q, Zhang C, Sui Y, et al. Development and validation of a hypoxia-related prognostic signature for breast cancer. Oncol Lett. 2020;20(2):1906-14.
28. Wang Z, Gao L, Guo X, Wang Y, Ma W, Guo Y, et al. A novel hypoxic tumor microenvironment signature for predicting the survival, progression, immune responsiveness and chemoresistance of glioblastoma: a multi-omic study. Aging (Albany NY). 2020;12(17):17038-61.
29. Karn T, Meissner T, Weber KE, Solbach C, Denkert C, Engels K, et al. A Small Hypoxia Signature Predicted pCR Response to Bevacizumab in the Neoadjuvant GeparQuinto Breast Cancer Trial. Clin Cancer Res. 2020;26(8):1896-904.
