# Supplementary material for: An Eight-Gene Hypoxia Signature Predicts Survival in Pancreatic Cancer and Is Associated With an Immunosuppressed Tumor Microenvironment
Source: Front Immunol. 2021 May 20;12:680435. doi: 10.3389/fimmu.2021.680435 (PMC8173254; doi:10.3389/fimmu.2021.680435)

Figure S1 Validating protein and gene expression in cell lines: (A) Immunoblots of HIF-1α protein expression in normoxic (N) and hypoxic (H) conditions in the fourteen cell lines. +: HIF-1α positive control. β-Actin: loading control. **(B)** Agarose gel electrophoresis of collected qPCR products from normoxic (N) and hypoxic (H) reactions for *18S* (187 bp), *ADM* (76 bp), *CA9* (78 bp), *LOX* (77 bp), *BNIP3* (125 bp) and *ANGPTL4* (92 bp), with a 100 bp ladder (L) as reference and *18S* as a loading control. **(C)** Median fold change of eight genes quantifiable in all cell lines reported as median ± 95% CI of three independent experiments from fourteen cell lines.

A

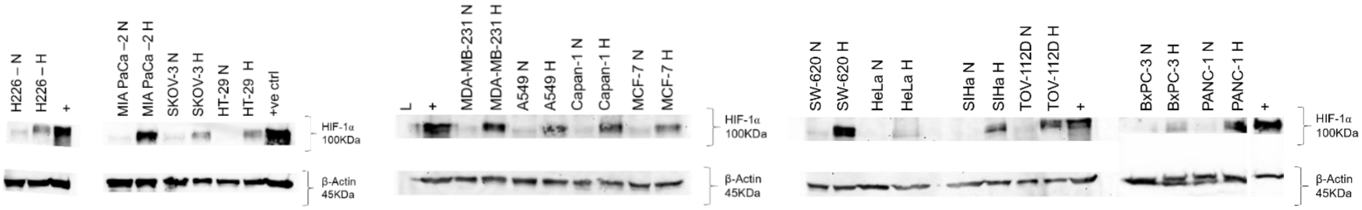

B

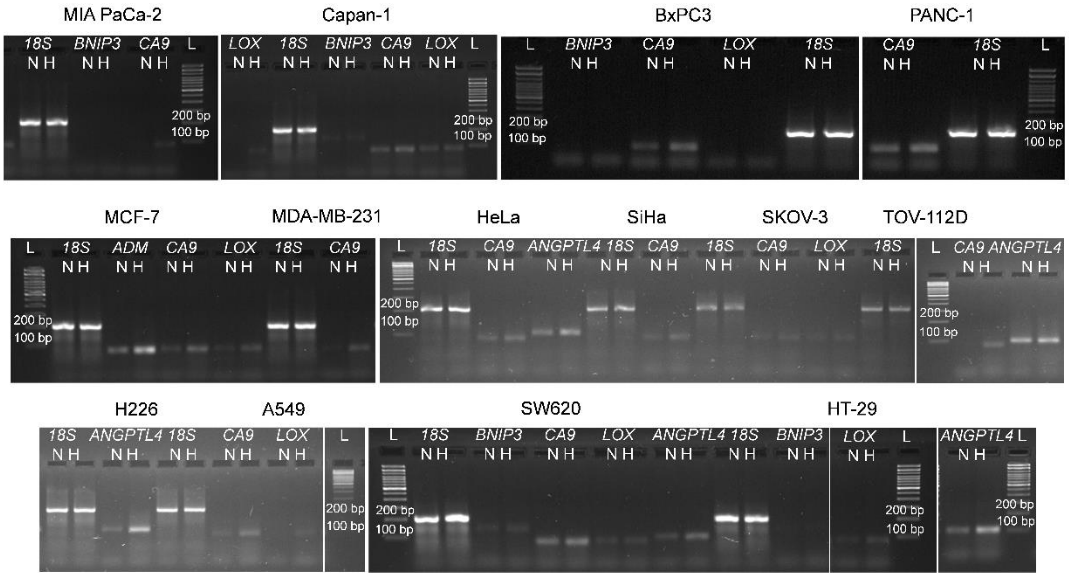

C

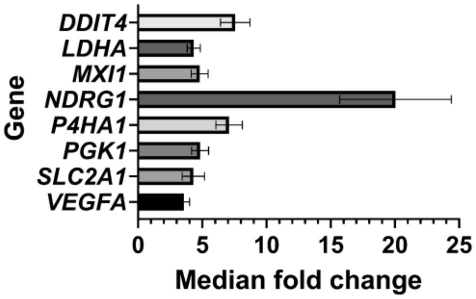

Figure S2 Validating signature in PDA datasets: (A) Expression of *ADAM10* (immune evasion gene), *CA9* (pH regulating gene), *ENO1*, *HILPDA* and *PGM2* (glycolysis genes) and *TWIST1* (EMT gene) in PAAD Data-1 hypoxia score (HS) high and low tumors. Significance is based on two-tailed t-test with p-value < 0.05 considered statistically significant. **(B)** Spearman correlation plots between the expression of the 8-gene hypoxia signature and hypoxia score. Strength of correlation reflected in the size of the colored square and white squares indicate no correlation present. **(C)** Kaplan-Meier survival plots with log-rank test, **(D)** ROC curve AUC analysis, and **(E)** Multivariate COX PH forest plots of hazard ratio, of the 8-gene hypoxia signature in HS high *vs* HS low tumors. **(A-C, E)** P-value < 0.05 considered statistically significant. FP: false positive (1 - specificity); TP: true positive (sensitivity).

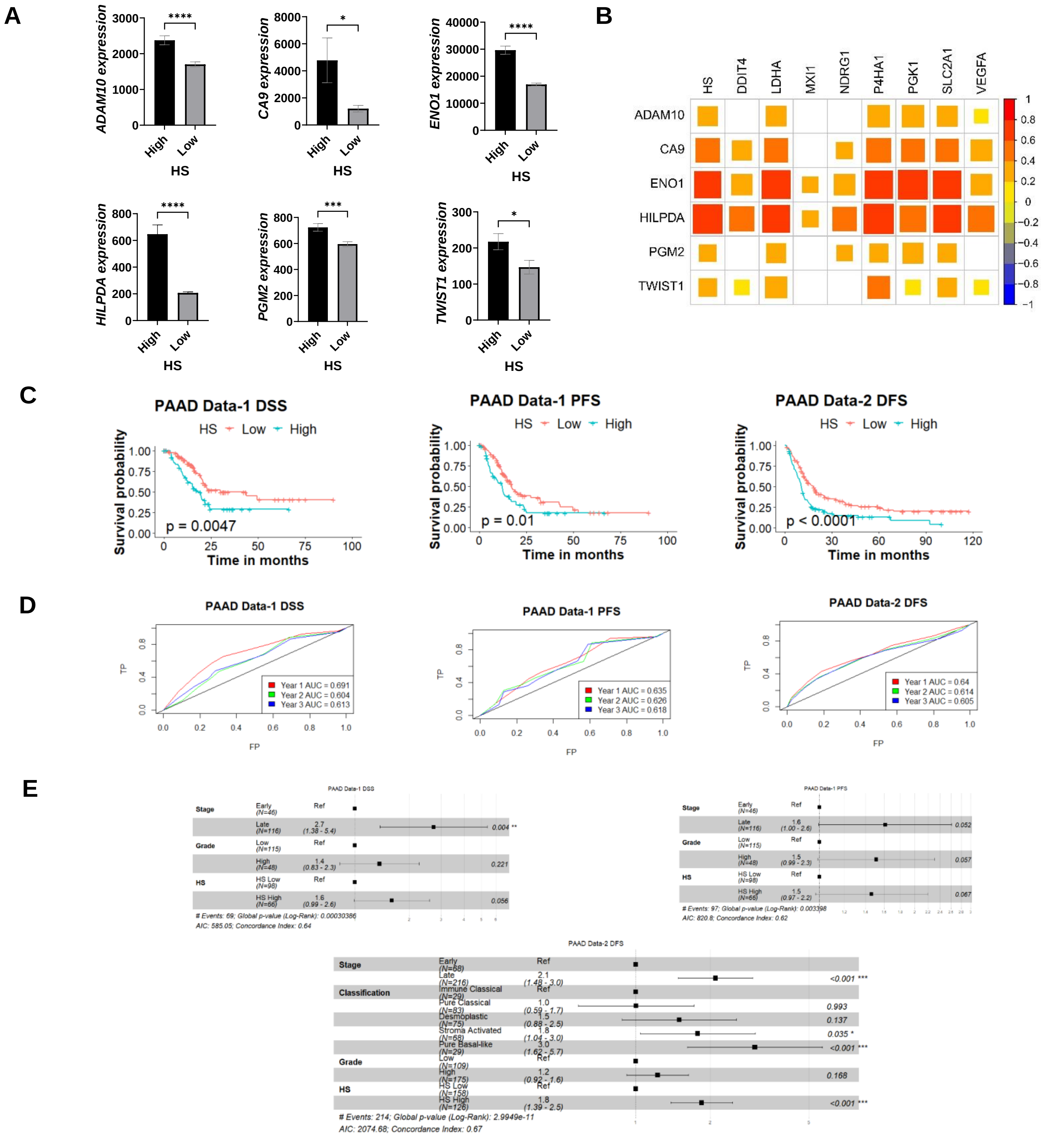

Figure S3 Evaluation of published hypoxia signature and tumor inflammation signature in PDA: (A) Forest plots comparing impact on hazard ratio of hypoxia score (HS) from the 8-gene signature and that from Li signature (Li) in multivariate COX PH analysis. **(B)** Kaplan-Meier survival plots with log-rank test comparing survival probabilities in IS (immune score) Low and IS High tumors.

A

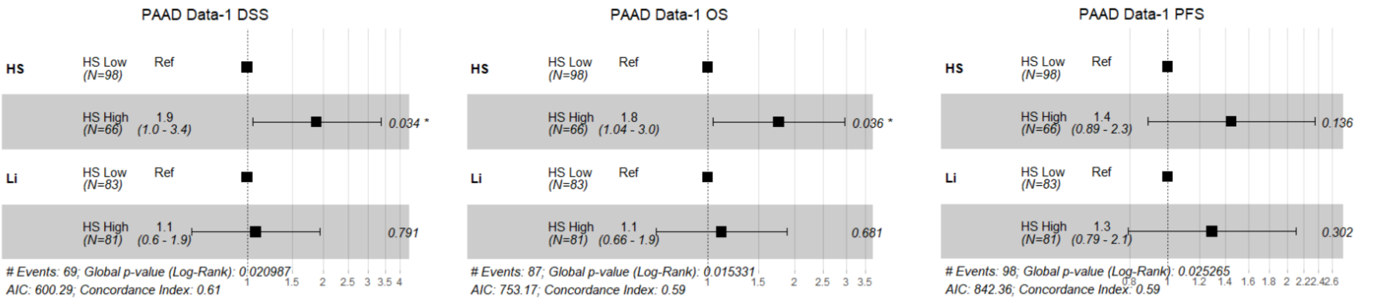

B

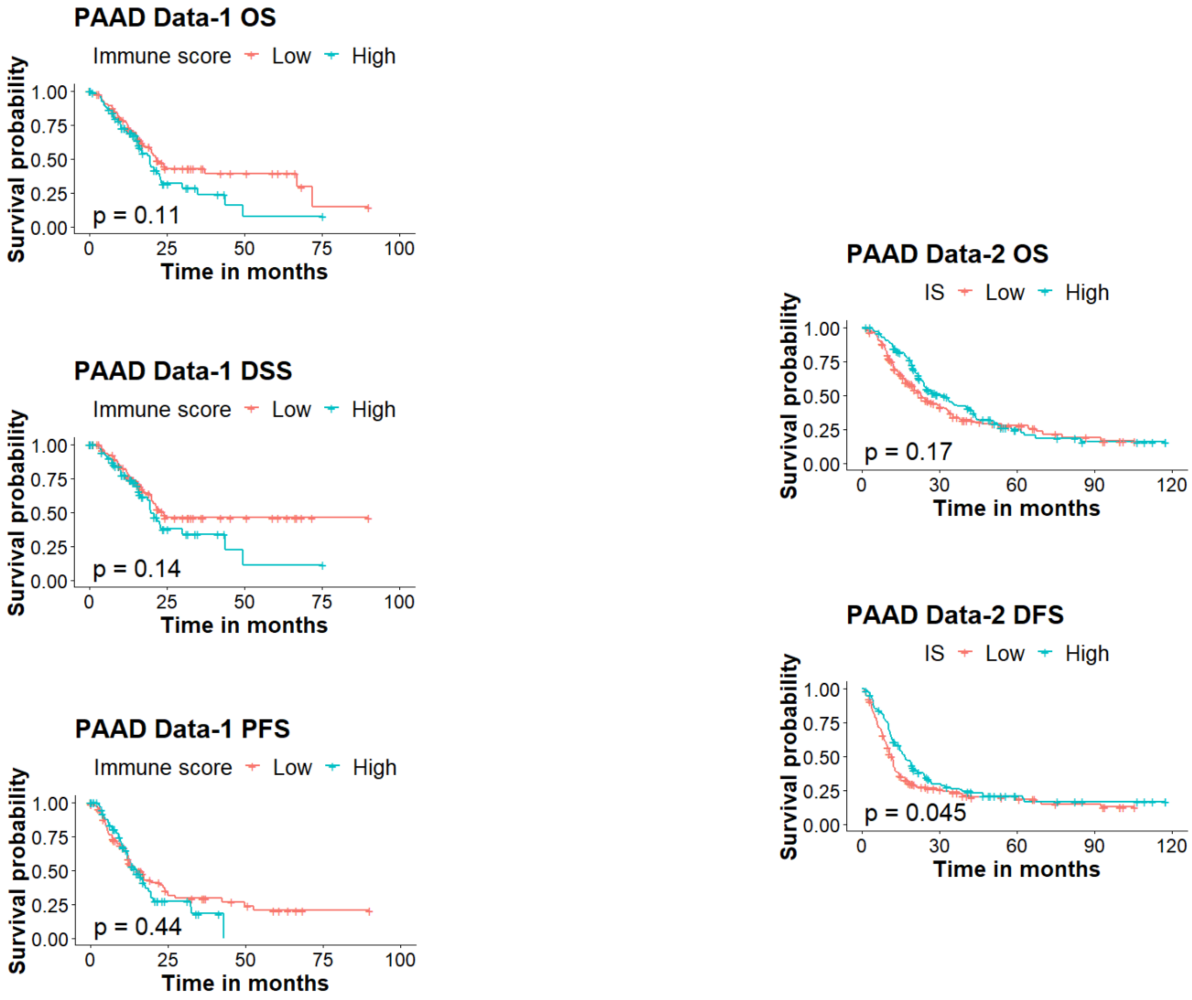

Figure S4 Validating a combined hypoxia-immune classification in PDA datasets: (A) Kaplan-Meier survival plots with log-rank test in the four indicated groups. **(B)** Kaplan-Meier survival plots with log-rank test, **(C)** ROC curve AUC analysis and, **(D)** multivariate COX PH forest plots of hazard ratio, of the 8-gene hypoxia signature in Hypoxia Low/Immune Low *vs* Hypoxia High/Immune low groups. P-value < 0.05 considered statistically significant. FP: false positive (1 - specificity); TP: true positive (sensitivity).

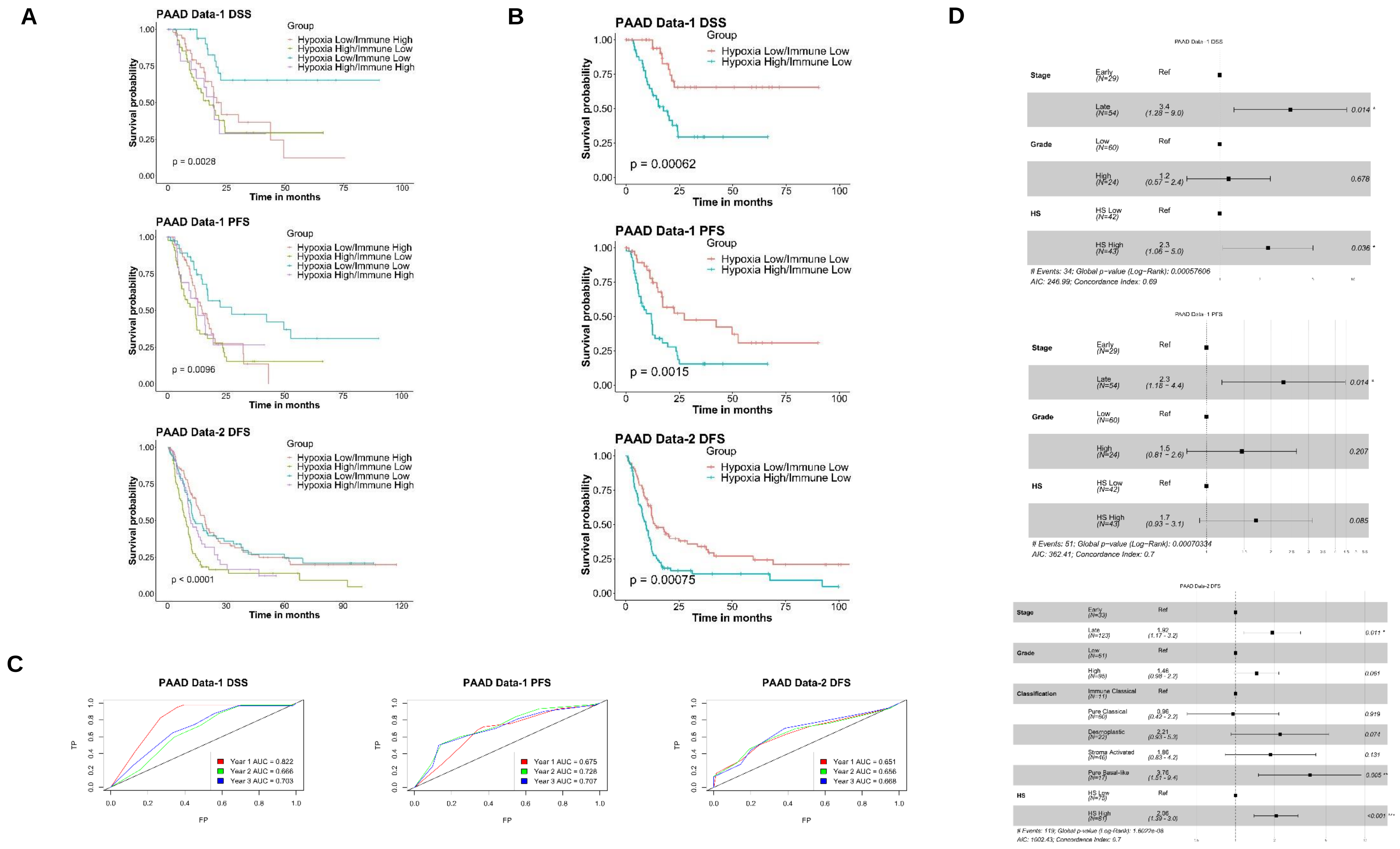

Supplement: Supplementary file 4 [file DataSheet_4.pdf]
